# Coordination Chemistry of Single‐Atom Catalysts in Expediating Kinetics for Advanced Lithium‐Sulfur Batteries

**DOI:** 10.1002/advs.78095

**Published:** 2026-10-20

**Authors:** Peng Chen, Jinyuan Zhao, Jing Zhang, Yidong Miao, Weihao Li, Huihua Li, Jian Wang, Yongzheng Zhang

**Affiliations:** ^1^ School of Textile and Clothing Nantong University Nantong China; ^2^ School of pharmacy Nantong University Nantong China; ^3^ School of Materials Science and Engineering Xi'an University of Technology Xi'an China; ^4^ School of Material and Chemical Engineering Xuzhou University of Technology Xuzhou China; ^5^ School of Chemistry University of Glasgow Glasgow UK; ^6^ Helmholtz Institute Ulm (HIU) Ulm Germany; ^7^ Karlsruhe Institute of Technology (KIT) Karlsruhe Germany; ^8^ Institute for Innovative Materials and Energy Faculty of Chemistry and Chemical Engineering Yangzhou University Yangzhou China

**Keywords:** catalytic efficiency, coordination chemistry, lithium sulfur battery, polysulfide conversion, single atom catalyst

## Abstract

The shuttling effect and sluggish conversion kinetics of lithium polysulfides (LiPSs) hinder the further advancement of lithium‐sulfur (Li‐S) batteries, exhibiting high barriers. Among catalysts, single‐atom catalysts (SACs) can accelerate LiPSs redox kinetics by leveraging M‐S coordination, interfacial charge transfer, and anchoring effect of coordinating heteroatoms. Numerous researches have reported improved catalytic activity via screening central metal and coordinating atoms as well as optimizing coordination configurations. However, systematic summary concerning the principles in screening and design of asymmetric coordination structures, the evolution mechanisms of orbital hybridization and spatial geometry, and the corresponding structure‐activity relationships governing LiPSs conversion kinetics remain unknown. In this review, we systematically summarize the modifications brought by asymmetric coordination chemistry and dual‐atom coordination structures in terms of geometric configuration engineering, molecular orbital hybridization, and interfacial charge rearrangement, together with the underlying mechanisms for boosting LiPSs conversion kinetics. Centered on theoretical calculations supplemented by experimental validations, we elaborate the critical significance of asymmetric coordination chemistry of SACs in elevating the catalytic efficiency. Finally, the future developments of SACs in Li─S batteries are outlooked and prospected.

## Graphical abstract

This review elucidates the catalytic mechanisms of single‐atom catalysts (SACs) in accelerating lithium polysulfides redox kinetics. Subsequently, the modifications brought by asymmetric coordination chemistry and dual‐atom coordination structures in terms of geometric configuration engineering, molecular orbital hybridization, and interfacial charge rearrangement are summarized. Finally, key design principles, future directions, and viable strategies are proposed.

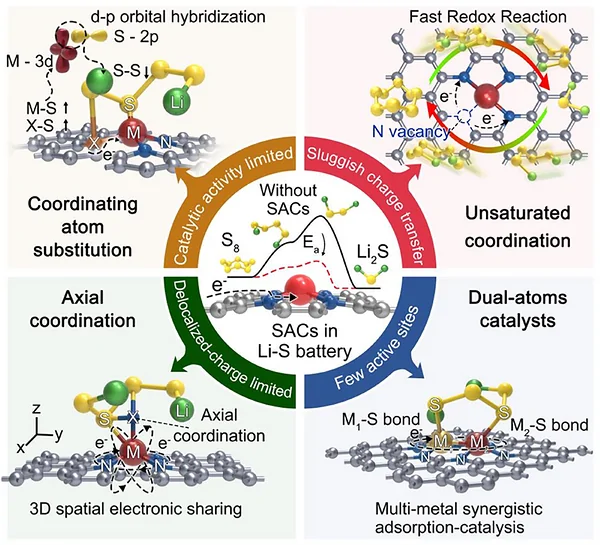

## 1 Introduction

Commercial lithium‐ion batteries (LIBs) suffer from low volumetric and gravimetric energy densities, which fail to meet the emerging market demands of artificial intelligence and economic applications [1, 2, 3, 4, 5]. Conventional cathode materials such as LiCO_2_ (LCO) and LiNi_x_Co_y_Mn_z_O_2_ (NCM) only exhibit low theoretical specific capacities within 300 mAh g^−1^, [5, 6, 7, 8] while, the redox conversions of elemental sulfur (S_8_) proceed via soluble lithium polysulfide (LiPSs, Li_2_S_n_, 4≤n≤8) and finally forms insoluble Li_2_S, which endows sulfur with an ultrahigh theoretical specific capacity of 1675 mAh g^−1^ [9, 10, 11]. When paired with Li metal anode, the theoretical energy density of lithium‐sulfur (Li‐S) batteries can reach as high as 2600 Wh kg^−1^ [12]. Furthermore, compared with high‐cost metals such as Ni and Co, sulfur boasts inherent merits of high natural abundance, low cost and environmental benignity, promising great potential for commercialization. However, the high energy barriers of S_8_ and Li_2_S induce severe electrochemical polarization and sluggish reaction kinetics, which severely limits the release of capacity in Li─S batteries [13, 14]. The energy barriers for the reduction of soluble Li_2_S_4_ to sparingly insoluble Li_2_S_2_ and further to Li_2_S are excessively high, making the conversion difficult (Figure 1a) [15]. More importantly, the reduction of Li_2_S_2_ to Li_2_S constitutes the rate‐determining step during discharge [16]. Compounding this kinetic bottleneck is the notorious LiPSs shuttling effect. The soluble long‐chain LiPSs generated in preceding steps cannot be fully consumed in time and readily dissolve in ether‐based electrolytes, and then diffusing through the separator toward the Li anode, where they are automatically reduced to Li_2_S [17, 18, 19]. This parasitic process not only causes irreversible loss of active sulfur but also leads to drastic attenuation of reversible capacity and inferior cycling stability, thereby undermining the long‐term reliability of Li─S batteries [20, 21].

**FIGURE 1:**
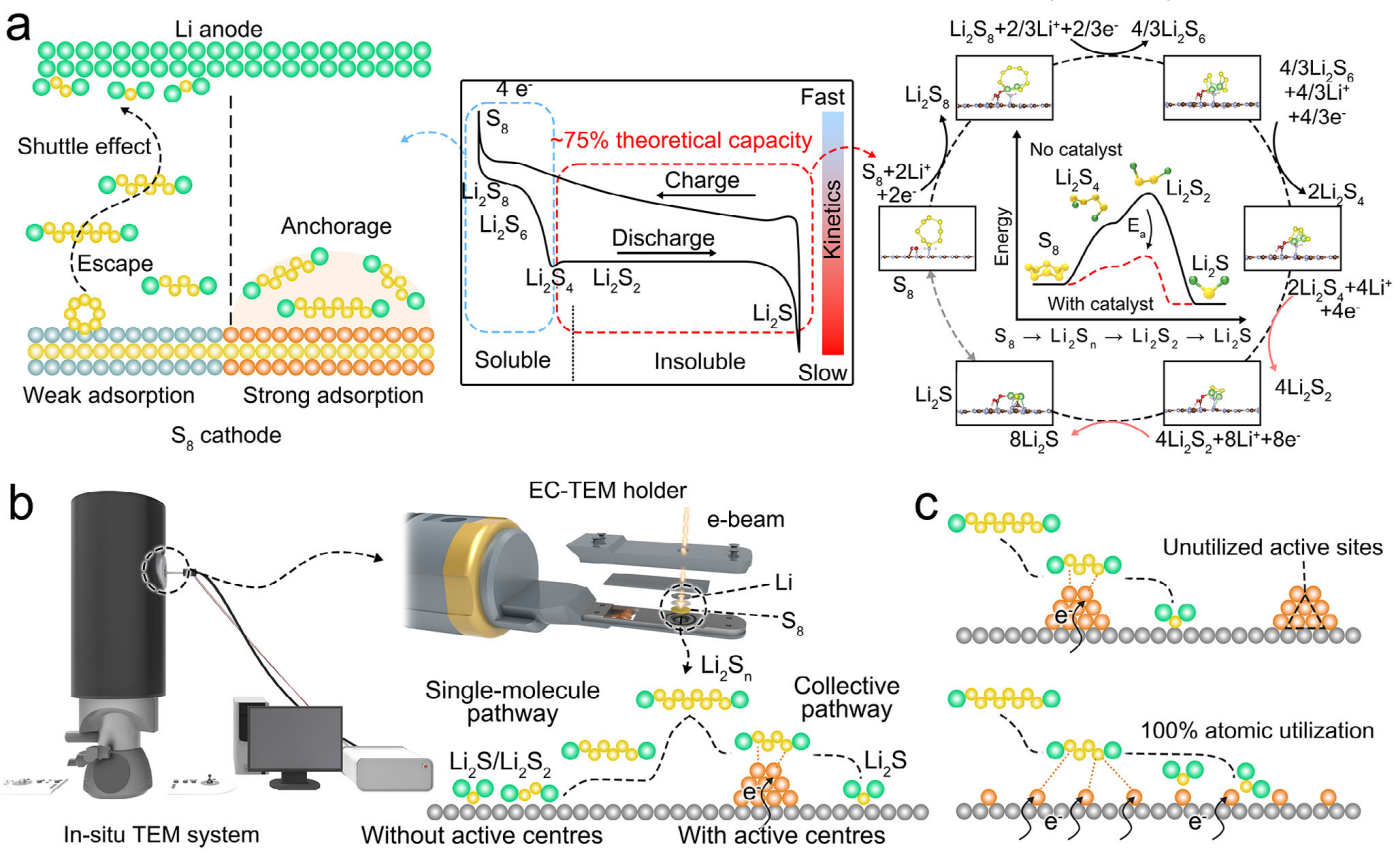
(a) Schematic illustration of LiPSs conversion mechanisms with/without electrocatalysts. (b) Schematic diagram for LiPSs conversion within/without the electrocatalysts. (c) Schematic illustration comparing the low active site utilization of nanoparticle catalysts and nearly full atomic utilization of SACs.

To address the sluggish conversion kinetics of LiPSs, various electrocatalysts have been incorporated either into the matrix host or as separator modifications, aiming to provide abundant active sites for capturing and immobilizing free LiPSs [22, 23, 24]. Moreover, the rapid charge transfer at these active sites lowers the energy barriers for LiPSs conversion, thus accelerating the overall redox kinetics (Figure 1a). For instance, Liao et al. adopted in‐situ transmission electron microscopy (in‐situ TEM) to directly visualize the strong interaction between metal active sites and LiPSs, expediting interfacial charge transfer and rapidly converting liquid LiPSs into metastable Li_2_S nanocrystals for uniform deposition (Figure 1b) [25]. Inspired by these mechanistic insights, researchers have recently designed and introduced metal carbides, oxides, nitrides, sulfides and their corresponding heterostructures. By modulating the charge environment around the central metal species, electrocatalysts can effectively boost the electrochemical performance of Li─S batteries [26, 27]. However, the stacking and agglomeration of nanoparticles render numerous active sites buried and inaccessible, thereby reducing the catalytic efficiency of electrocatalysts (Figure 1c) [28, 29, 30].

Single‐atom catalysts (SACs) offer the distinctive advantage of nearly 100% atomic utilization efficiency [31, 32, 33, 34]. Combined with their tunable central metal atoms, diverse ligands, and flexible geometric configurations, SACs have attracted extensive research interest in recent years [35, 36]. Compared to conventional nanoparticle‐based catalysts, SACs feature prolonged catalytic lifespans, superior selectivity and lower manufacturing costs, offering a cost‐effective and high‐efficiency strategy for the practical deployment of Li─S batteries. To date, more than 30 types of SACs have been demonstrated as viable central metal active sites (Figure 2a) [37]. In the context of Li─S batteries, Fe‐, Co‐ and Ni‐based SACs are the most widely investigated [38, 39, 40]. A broad variety of coordinating ligands have also been reported in the literature, encompassing elements such as C, N, O, P and S [41]. Meanwhile, diverse carbonaceous supports are adopted, including graphene, N‐doped carbon, and metal‐organic framework (MOF)‐derived carbon. Moreover, the introduction of electron‐rich transition metals with unfilled *d*‐orbitals, such as Fe, Co, Ni, facilitates the adsorption of polar LiPSs, strengthens M‐S bonding and elongates S‐S bond lengths (Figure 2b). In addition, *d‐p* orbital hybridization occurs between the *d* orbitals of the metal centers and the *p* orbitals of coordinating nonmetallic atoms (N, O, etc.), triggering localized charge transfer [42]. This electronic modulation accelerates efficient electron transfer to the adsorbed LiPSs, thereby promoting the redox kinetics of sulfur conversion. In our previous research, we combined Co SACs with the Li_2_S cathode via thermal treatment to achieve long‐cycle stability of Li─S batteries (441 mAh g^−1^ at 2.0 C) [43]. It is demonstrated that the incorporation of Co atoms effectively rearranges the local charge distribution of the graphene substrate, elevates the density of states near the Fermi level, accelerates charge transfer, reduces the conversion energy barriers of LiPSs (1.34 vs. 2.14 eV) and the diffusion barrier of Li (0.089 vs. 0.254 eV), facilitating the conversion from Li_2_S to Li_2_S_n_ (Figure 2c). Meanwhile, the formation of Co─S bonds strongly immobilizes Li_2_S_4_ with high binding energy (2.90 eV), thus suppressing the shuttling effect of Li_2_S_n_ (Figure 2d). Besides, we have also verified that nano‐sized Fe sites exhibit strong adsorption capacity toward long‐chain Li_2_S_n_ to inhibit the shuttling effect. Accordingly, atomically dispersed Fe SACs were immobilized on porous carbon was been reported [44]. The synergistic anchoring effect of Fe and N sites together with the high catalytic activity of SACs reduces the decomposition barrier of Li_2_S from 3.4 to 0.81 eV and lowers the overpotential of Li_2_S oxidation (Figure 2e). Moreover, Fe SACs were integrated into Fe_3_C nanoparticle modified porous carbon (SAPTC@PCS) [45]. Benefiting from highly active Fe─N_4_ SACs and intense interfacial charge transfer between Fe─N_4_ and Fe_3_C, the delocalized charge sharing radius expands, decreasing the decomposition barrier of Li_2_S_n_. More importantly, simulations demonstrate that Fe─N_4_ disrupts the Li(solvents)_x_^+^ clusters to release abundant free Li^+^, thus accelerating the conversion of Li_2_S_n_ with a reduced barrier of 1.17 eV [46]. Based on these findings, we also extended SACs engineering in Li metal batteries. By using the modulation of desolvation barrier of Li(solvents)_x_^+^ and the Li^+^ diffusion barrier realizes optimized Li^+^ diffusion/nucleation kinetics. In our prior research, Co SACs were implanted into defective Fe_1‐x_S to obtain SACo/ADFS@HPSC with vacancies and SACs sites, synergistically inducing uniform Li nucleation and diffusion [47]. Thus the SACo/ADFS@HPSC based Li||Li symmetric cell delivers stable galvanostatic Li plating/stripping for 1600 h, while the blank sample only lasts 500 h, which offers a novel strategy for constructing high‐energy‐density Li metal batteries. Nevertheless, in previous work, we only elucidated the atomic‐scale effects of SACs on the conversion energy barriers of LiPSs and the evolution of Li(solvents)_x_^+^ structures from the perspectives of molecular dynamics and reaction thermodynamics. There remains a lack of deeper level insights. For instance, from the perspective of orbital hybridization into how the interactions between SACs and LiPSs modulate sulfur redox kinetics. Furthermore, whether optimizing the coordination configuration of SACs can further boost the catalytic activity of SACs remains unclear, which had been overlooked in our previous work.

**FIGURE 2:**
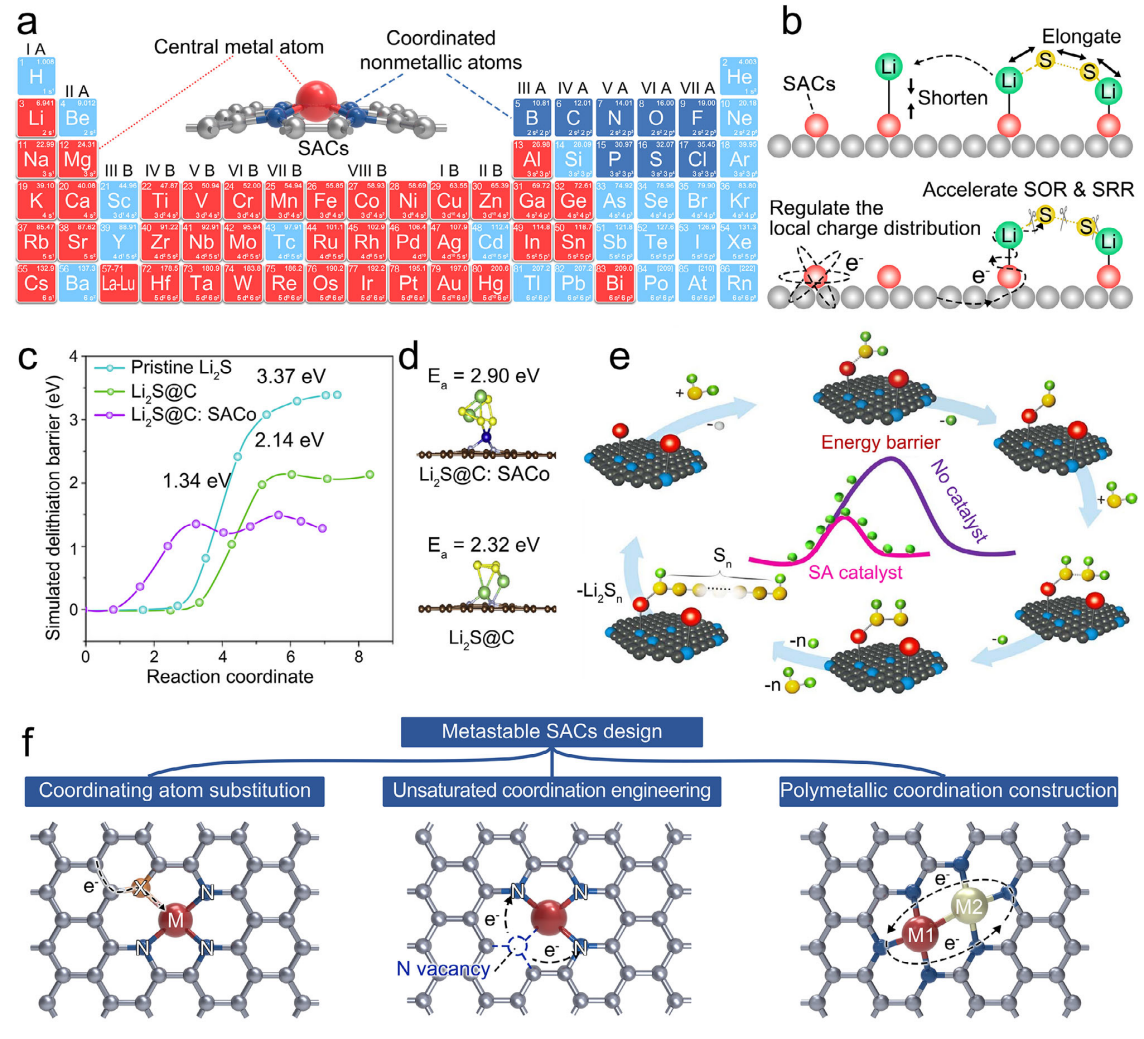
(a) Schematic diagram for atomic screening of SACs. (b) Schematic diagram for accelerated LiPSs redox on the SACs. (c) Comparison of simulated delithiation energy barrier of Li_2_S. (d) Adsorption energy of Li_2_S_4_ on NC with/without Co SACs. Reproduced with permission [43]. Copyright 2020, Elsevier B.V. (e) A proposed mechanism for SACs catalyzed Li_2_S delithiation reaction. Reproduced with permission [44]. Copyright 2019, Elsevier B.V. (f) Design logic of metastable SACs coordination structure.

As we know, the intrinsically stable symmetric M─N_4_ coordination structure inherently limits the catalytic activity of single‐atom active sites from being fully exploited [48]. To overcome this limitation, catalytic centers with metastable configurations can be constructed by adjusting the type and number of coordinating ligands or by fabricating polymetallic active sites [49, 50]. These strategies trigger local charge redistribution and generate an inhomogeneous local electric field, thereby further boosting the intrinsic catalytic activity of SACs. From the perspective of coordination chemistry, the conventional planar symmetric M─N_4_ configuration corresponds to the global thermodynamic steady state with saturated coordination, minimized system energy and a highly stabilized electronic structure. In contrast, metastable coordination configurations are defined as kinetically stabilized, thermodynamically excited active configurations constructed, including unsaturated coordination, heteroatom coordination, axial coordination, and dual‐atom catalyst designs [51]. Thermodynamically, the metastable coordination configurations elevate the overall electronic energy of metal centers. Such high‐energy coordination states are thermodynamically unstable and intrinsically tend to reconstruct toward the stable M─N_4_ structure, endowing the sites with thermodynamic metastability [52, 53]. From a kinetic perspective, although metastable configurations possess higher free energy, they are also trapped and stabilized by a high structural reconstruction energy barrier. More specifically, the rigid carbon substrate and multi‐ligand bonding confinement effectively suppress spontaneous structural collapse, metal migration, and aggregation under electrochemical operating conditions. Therefore, metastable coordination configurations maintain long‐term structural viability during cycling, exhibiting the unique feature of thermodynamic instability but kinetic persistence. Moreover, from the orbital coordination perspective, the saturated planar M─N_4_ structure exhibits highly symmetric orbital hybridization, which strongly confines metal 3d orbitals, reduces electronic state density near the Fermi level, and limits *d‐p* orbital overlap between metal and sulfur species [54, 55]. In comparison, metastable coordination breaks the orbital symmetry of M─N_4_, reorganizes the energy level distribution of metal d‐orbitals, generates abundant unsaturated frontier orbitals, and promotes efficient d‐p orbital hybridization. Such orbital rearrangement not only accelerates interfacial charge delocalization and transfer kinetics but also optimizes the adsorption strength of LiPSs by modulating orbital filling and antibonding state occupation. Accordingly, the metastable orbital coordination environment fundamentally eliminates the orbital confinement limitation of conventional M─N_4_‐based SACs, thus enhancing the LiPSs catalytic conversion. With the aim of advancing the practical application of SACs in Li─S batteries, this review systematically elaborates the interactions among metal orbitals, coordination environments, structural modulation and LiPSs behavior, from multiple design perspectives including central metal selection, coordinating atom optimization, unsaturated coordination engineering to polymetallic coordination construction (Figure 2f). On this basis, the core design principles of SACs for Li─S batteries are clarified, and prospective research directions are also outlined.

## 2 Designs of Metastable Coordination Structure for SACs

The outstanding activity and selectivity of SACs rely on the rational design and optimization of key factors, including electronic configuration, coordination environment, morphology and microstructure [56]. Although planar symmetric coordination structures can guarantee structural stability and extend the service life of SACs, the symmetric Metal─N_4_ coordination configuration imposes constraints on the local electronic bound states, inhibiting electron delocalization and sharing, thus leading to sluggish charge transfer between SAC active sites and LiPSs, ultimately resulting in incomplete conversion of LiPSs [57, 58].

### 2.1 Coordinating atom Substitution on Matrix

Substituting the N atoms with other non‐metallic atoms breaks the symmetry of the M─N_4_ configuration and constructs metastable M─N_z_X structures (X = C, O, P, S, etc) [59, 60]. The resulting asymmetric coordination and electronegativity disparities induce uneven electron distribution, strengthen electron‐hole separation, and further boost the catalytic reactivity of the central metal sites (Figure 3a) [61, 62, 63]. Moreover, optimizing the coordinating atoms modulates the *d*‐orbital filling degree and electron spin state of the central metal, which improves the charge transfer efficiency between transition metals (TMs) and sulfur species. What's more, the number of valence electron, electronegativity and atomic radius of the nonmetallic coordinating atoms can significantly influence the electron density distribution and geometric configuration of the central metal atoms [64]. Sun et al. constructed theoretical models by substituting N sites in M─N_4_ with a series of heteroatoms, including S, P, B, Se, and Te, to fabricate asymmetric Co─N_3_X coordination structures, and systematically elaborate the effects of heteroatom coordination on catalytic performance [65]. Heteroatom coordination triggers structural distortion of the Co─N_3_X configuration, which narrows the width of the *d*‐band and shifts the *d*‐band center upward. Among the investigated heteroatoms, the incorporation of B reduces the electron occupation of antibonding orbitals and strengthens the *d‐p* orbital hybridization between SACs and LiPSs, as further confirmed by the integrated crystal orbital Hamiltonian population (ICOHP) values. As a result, Co─N_3_B delivers the strongest binding energy toward Li_2_S_4_ at 1.65 eV (Figure 3b). In comparison, the introduction of P and S moderately enhances the adsorption energy of Co─N_3_X for LiPSs [66]. In contrast, heteroatoms with larger atomic radii, such as Se and Te, may induce excessive structural distortion and even structural collapse, leading to weakened adsorption energy. The adsorption performance of Co─N_3_Se or Co─N_3_Te still surpasses that of the conventional M─N_4_ configuration. Moreover, replacing N (5 e^−^) with B (3 e^−^), which possesses a larger atomic radius, creates a local electron‐deficient region, accelerating the separation of local electrons and holes, and improving the catalytic activity of Co SACs. Thus, the Gibbs free energy for the conversion of Li_2_S_2_ into Li_2_S on Co─N_3_B is reduced from 0.74 to 0.66 eV. Furthermore, Li_2_S nucleation experiments verify that Co─N_3_B facilitates instantaneous 3D deposition of Li_2_S, preventing the coverage of catalytic active sites and sustaining long‐term high catalytic activity (1038.6 mAh g^−1^ at 0.2 C). Moreover, constructing metastable Co─N_3_B configurations via B‐doping lowers the electron occupation of antibonding orbitals, optimizes the *d‐p* orbital hybridization between the central Co atoms and sulfur species, and thus accelerates the redox kinetics of LiPSs (Figure 3c) [67].

**FIGURE 3:**
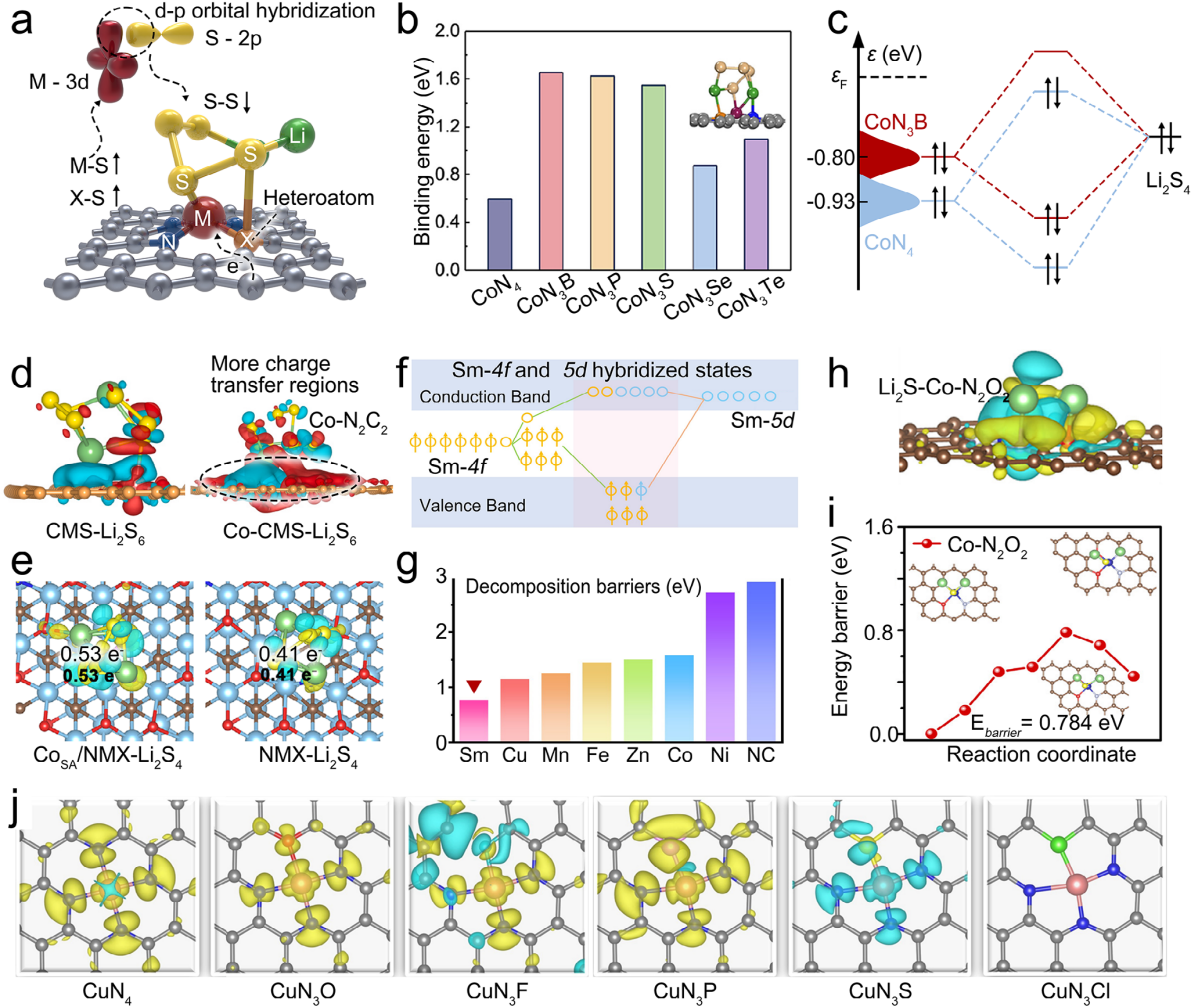
Mechanism diagram for LiPSs redox on the M─N_3_X. (b) Binding energy of Li_2_S_4_‐Co─N_3_X. (c) Scenario of d‐p orbital hybridization for Li_2_S_4_‐Co─N_3_B and Li_2_S_4_‐Co─N_4_. Reproduced with permission [65]. Copyright 2024, Elsevier B.V. (d) Electron density difference of Li_2_S_6_‐CMS with/without Co─N_2_C_2_. Reproduced with permission [69]. Copyright 2025, Elsevier B.V. (e) Electron density difference. Reproduced with permission [71]. Copyright 2025, Wiley‐VCH GmbH. (f) Schematic diagram of mott transition of electrons from 4f to 5d‐orbitals. (g) Decomposition energy barriers of Li_2_S on SACs. Reproduced with permission [72]. Copyright 2024, Wiley‐VCH GmbH. (h) Electron density difference of Li_2_S‐Co─N_2_O_2_. (i) Decomposition energy barriers of Li_2_S on Co─N_2_O_2_. Reproduced with permission [73]. Copyright 2024, Elsevier B.V. (j) Electron density difference of M─N_3_X. Reproduced with permission [75]. Copyright 2025, American Chemical Society.

Heteroatom substitution can significantly induce asymmetric local charge distribution, and the differences in electronegativity and atomic radius further modulate the catalytic activity of central metal atoms [68]. Chen et al. designed a Co─N_2_C_2_ coordination structure to optimize the electronic configuration of the central Co atoms, which boosts electron transfer between Co and LiPSs [69]. Since C has a lower electronegativity (2.55 eV) than N (3.04 eV), more substrate defects can be generated, and the electron density shifts toward N atoms with expanded charge distribution regions, reducing the Gibbs free energy for the conversion of Li_2_S_2_ to Li_2_S on Co─N_2_C_2_ (from 1.43 to 0.43 eV), which greatly accelerates the redox kinetics of LiPSs (Figure 3d). Yin et al. disrupted the stable Fe─N_4_ coordination to construct metastable Fe‐C_2_N_2_ coordination structures, reducing the electron occupation of the antibonding orbitals of Fe, reinforcing the interaction between Fe─N_2_C_2_ and LiPSs, and accelerating the reduction of Li_2_S [70]. Meanwhile, Sinthika et al. further verified that the average length of M‐N bonds gradually shortens with increasing coordination number of N substituting C sites, while the structural stability of SACs first rises and then declines [68]. This phenomenon originates from the repulsive interaction between adjacent N atoms and the shift of electron polarization induced by electronegativity differences. Apart from the modulation of catalytic activity originating from varied coordinating atoms, the orbital electron occupation of the central metal atoms also exerts a significant influence on electrocatalytic performance. For instance, metals with half‐filled *d* orbitals such as Fe, Co and Ni, exhibit invariant occupation of the d_z_^2^ orbital. In contrast, metals with low *d*‐orbital filling (Ti, V, Cr) show dramatic fluctuations in d_z_^2^ orbital occupation as the N coordination number changes, accompanied by corresponding variations in the occupation of d_xz_ and d_yz_ orbitals. Theoretical calculations further confirm that the optimal binding energy between SACs and Li_2_S ranges from ‐2.5 to ‐4.0 eV to achieve uniform Li_2_S deposition and prevent active site poisoning. Modulating the substrate material to construct single‐atom active sites with distinctive coordination modes is another effective strategy. Ma et al. anchored Co SACs on N‐doped MXene substrates to fabricate an asymmetric Co─N_1_C_3_ coordination structure (CoSA‐CoNC/NMX), which triggers local structural distortion accompanied by obvious bond length contraction [71]. Meanwhile, the asymmetrically coordinated Co SACs shift the d‐band center of Ti in MXene upward from ‐0.88 to ‐0.34 eV. Compared with N, elemental carbon creates an electron‐deficient state on the central Co atoms, which strengthens the adsorption affinity toward LiPSs. In the optimized Li_2_S‐CoSA‐CoNC/NMX theoretical model, the bond lengths of Li_1_‐S and Li_2_‐S stretch to 2.118 and 2.148 Å, respectively, due to the strong interaction between Co and S. Moreover, Co─N_1_C_3_ donates more electrons to Li_2_S_4_ (0.53 e^−^ vs. 0.41 e^−^), lowering the decomposition energy barrier of Li_2_S on CoSA‐CoNC/NMX to 1.01 eV, lower than that of bare NMX (1.26 eV, Figure 3e).

The orbital hybridization between the *3d*‐orbitals of metal sites (Fe, Co, Ni, etc.) and the *2p*‐orbitals of S facilitates rapid charge transfer. Nevertheless, SACs constructed from 3d‐orbitals metals tend to suffer from failure during long‐term cycling. Rare‐earth metals, with vacant 5d‐orbitals and electron‐rich 4f orbitals, can strengthen the interaction with S *2p* and boost the amount of charge transfer between the SACs and LiPSs. Specifically, Gu et al. synthesized rare‐earth‐based SACs featuring Sm─N_3_C_3_ coordination, and realized rapid conversions of LiPSs via 4f‐5d‐2p orbital coupling [72]. Figure 3f reveals that within the Sm─N_3_C_3_ coordination configuration, the electronic state of Sm transforms from 4f^6^5d^0^ to 4f^5^5d^1^, generating f‐d orbital hybridization. Meanwhile, Mott transition of electrons from 4f to 5d‐orbitals raises the density of electronic states in the originally empty 5d‐orbitals, which further facilitates electron transfer to the *2p*‐orbital of S. In addition, 4f‐5d‐2p orbital coupling narrows the d‐band center of Sm, further verifying the accelerated interfacial charge transfer kinetics. Consequently, the decomposition barrier of Li_2_S on Sm─N_3_C_3_ (0.72 eV) is much lower than those of Fe─N_4_/C (1.44 eV), Co─N_4_/C (1.58 eV) and Ni─N_4_/C (2.72 eV, Figure 3g).

To break the equilibrium state of M─N_4_ and induce localized electron polarization, Li et al. substituted an N coordinating atom (χ_N_ = 3.04) with a highly electronegative O atom (χ_O_ = 3.44) and fabricated SACs with strongly polar Co─N_2_O_2_ coordination configurations [73]. Bader charge calculations reveal that the high electronegativity of O enables the formation of stronger chemical bonds, realizing effective anchoring of LiPSs with the synergistic effect of Co‐S and Li‐O bonds (Figure 3h). Furthermore, the electronegativity difference between O and N draws more free electrons from the central Co atom toward the coordinating heteroatoms, exacerbating the unsaturated coordination state of Co and further strengthening the Co‐S binding energy [74]. In the symmetric Co─N_4_ coordination structure, Co serves as the catalytic active center, with a reaction energy barrier of 1.067 eV for Li_2_S conversion. In contrast, the introduction of asymmetric coordination and highly electronegative O atoms increases the charge transfer quantity between Li_2_S_n_ and Co─N_2_O_2_ to 0.772 e^−^, while the reaction energy barrier of Li_2_S on Co─N_2_O_2_ decreases to 0.784 eV (Figure 3i). Ex‐situ X‐ray photoelectron spectroscopy (XPS) characterizations demonstrate that Li atoms spontaneously coordinate and anchor to the N sites of Co─N_2_O_2_ during discharge, and the Co‐O bonds simultaneously accelerate interfacial electron transport. In addition, N 1s shifts are observed in the XPS peaks before and after electrochemical reactions, which convincingly verifies the robust structural stability of the SACs.

Apart from conventional coordinating heteroatoms including B, C, N and O, F possesses the highest electronegativity (χ_F_ = 3.98) and the smallest atomic radius (71 pm), which can induce pronounced electron polarization, expanding the regions of charge accumulation and charge dissipation, thereby elevating the catalytic activity of SACs. Xu et al. replaced N coordination sites with various heteroatoms to construct asymmetric Cu─N_3_X coordination structures, which trigger geometric Jahn–Teller distortion [75]. Optimized structural models indicate that the incorporated F atom can form covalent bonds with the C skeleton, weakening the binding force between the coordination atoms and the central Cu atom. Moreover, unlike the strong electron‐withdrawing effect of N and O, F generates a moderate negative charge at the C framework, establishing favorable charge transfer channels that facilitate electron migration from Cu─N_3_F_1_ to adsorbed Li_2_S (Figure 3j). As a result, the anchored Li_2_S carries the largest positive charge (0.71 e^−^). While, the moderate electron‐withdrawing character of F maintains a proper charge density on the central Cu atom, avoiding excessive electron depletion or insufficient electron polarization. Specifically, Cu─N_3_Cl exhibits defective spin states in the electronic configuration of Cu, which reduces the number of free electrons [76]. In contrast, Cu─N_3_F undergoes obvious Jahn‐Teller distortion and delivers a strong spin magnetic moment, accelerating the rapid conversion of LiPSs. Accordingly, Cu─N_3_F displays an ultralow reaction energy barrier (0.19 eV), followed by Cu─N_3_O (0.43 eV) and CuN_3_Cl (1.09 eV). However, the incorporation of B and C atoms with lower electronegativity and few valence electrons, tends to reduce the local free‐electron density, potentially giving rise to electron‐deficient regions that impede local charge transport. In contrast, the introduction of coordinating atoms with high electronegativity, such as O and F, can induce a localized distortion of the electric field, leading to electron localization and consequently diminishing the overall catalytic activity. To address this issue, it is advisable to introduce coordinating atoms with weakly electronegative and riched valence electrons, such as P and S. Which not only increases the number of free electrons but also promotes electron delocalization and facilitates local charge transfer, thereby accelerating the breakage of S─S bonds.

P (χ_P_ = 2.19) has a lower electronegativity than C (χ_c_ = 2.55) and N. Accordingly, substituting N atoms with P atoms or doping P into C hexagonal ring enable the diffusion of localized electrons and the formation of charge‐sharing regions (Figure 4a). Meanwhile, the atomic radius of P (110 pm) is larger than those of C (77 pm) and N (75 pm), which facilitates orbital hybridization of sp^3^ and local coordination structural distortion, thus broadening the 3D electron delocalization range and improving the catalytic activity of SACs (Figure 4a). Tang et al. screened a series of TM@N_3_P_1_ SACs (TM = Sc, Ti, V, Cr, Mn, Fe, Co, Ni, Cu, Zn), and verified that N_3_P_1_ coordination structure exhibits lowest energy barriers for LiPSs conversion and the lowest Li_2_S nucleation overpotential, especially Sc@N_3_P_1_ (1.22 eV, 0.19 V, Figure 4b) [77, 78]. Furthermore, Sc possesses a higher coordination binding energy with the N_3_P_1_ substrate, favoring robust structural stability, whereas Zn shows weaker binding affinity toward N_3_P_1_ [79]. Moreover, the Sc‐P bond is slightly stronger than the Sc─N bond, which promotes electron delocalization. Upon Li_2_S adsorption on Sc sites, the P─S bonds form in addition to intrinsic Sc─S bonds, and abundant lone pair electrons transfer from P to S centers to accelerate the breaking of Li─S bonds. Further investigations reveal that P incorporation induces a more intricate electron density distribution over TM@N_3_P_1_, which evolves the catalytic active center from an isolated central metal atom to a synergistic dual‐site system consisting of TM and P atoms [80]. Nevertheless, the overall catalytic performance is governed by the magnitude of charge transfer between the central metal and P atoms. For instance, the density functional theory (DFT) calculations demonstrate that V@N_3_P_1_ presents the lowest conversion energy barrier (0.79 eV). However, the V center loses excessive electrons, generating an overly strong adsorption affinity toward sulfur species and triggering catalyst deactivation during prolonged cycling. In contrast, Sc@N_3_P_1_ achieves superior electrochemical performance via multi‐bond interactions among Sc, P and S, featuring a high Li_2_S nucleation potential (2.23 V) and a moderate LiPSs conversion barrier (1.22 eV), as shown in Figure 4c. Ji et al. substituted N with P atoms to break the intrinsic symmetric Ni─N_4_ coordination structure and modulate the electronic properties of central Ni atoms, which shifts the d‐band center of Ni atom toward the Fermi level (Figure 4d) [81]. Accordingly, the valence state of Ni in Ni─N_3_P decreases to +1.20, compared with +1.76 in Ni─N_4_, which endows central Ni atoms with abundant free electrons available for transfer to LiPSs. Furthermore, substituted N with P atom at Ni─N_4_ sites triggers local structural distortion, which increases the Ni‐N bond length from 1.32 Å to 1.40 Å and forms Ni‐P bonds (∼1.70 Å). Theoretically, moderate bond elongation weakens the strong electronic confinement from planar N coordination atom, delocalizes metal *d‐*orbitals, and improves spatial orbital matching between metal *d*‐orbitals and sulfur *p*‐orbitals, thus modulating *d‐p* hybridization strength for LiPSs. Bader charge analysis further corroborates that the electron transfer quantity between Li_2_S_4_ and Ni─N_4_ rises from 0.04 e^−^ to 0.5 e^−^ for Ni─N_3_P. Meanwhile, theoretical optimization reveals that unlike the original Ni─N_4_ configuration where electron transport solely occurs between Ni and S, new P─S bonds form in Ni─N_3_P, enabling more effective anchoring of LiPSs. As a result, the Ni─N_3_P based Li||S cell retains a high capacity retention of 92% after 200 cycles at 0.2 C under a high sulfur loading of 6 mg cm^−2^.

**FIGURE 4:**
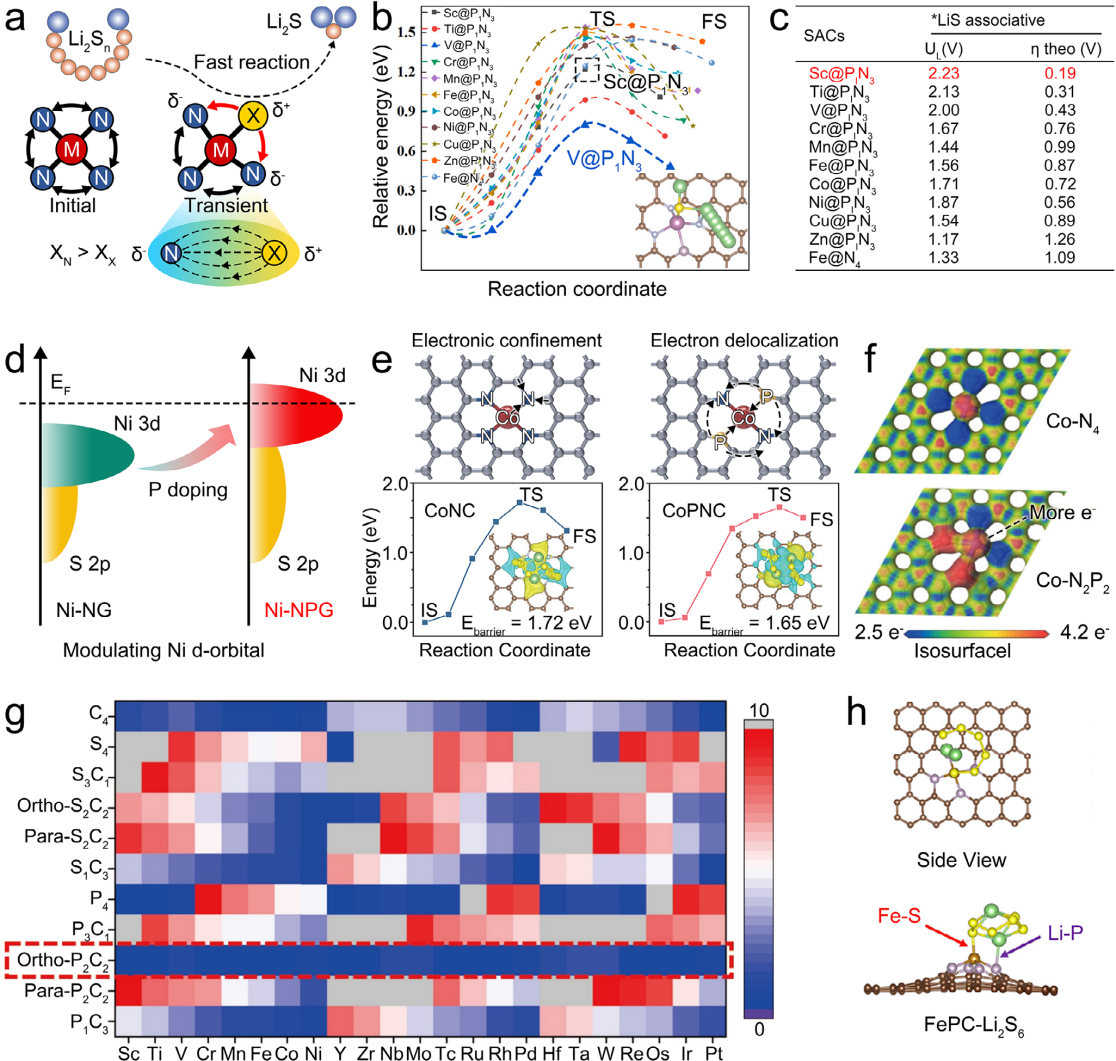
(a) Mechanism diagram for accelerated sulfur redox by weakly electronegative coordination atoms. (b) Relative energy of Li_2_S conversion on the TM@N_3_P_1_. (c) Theoretical overpotentia and ultimate potential values of TM@N_3_P_1_ applied in Li_2_S_2_ reduction. Reproduced with permission [78]. Copyright 2024, Elsevier B.V. (d) Schematic of the Ni d‐orbitals near the Fermi level and the S p‐orbitals structure. Reproduced with permission [81]. Copyright 2025, Wiley‐VCH GmbH. (e) Decomposition energy barrier of Li_2_S conversion on the Co─N_4_ and Co─N_2_P_2_. Reproduced with permission [86]. Copyright 2024, Wiley‐VCH GmbH. (f) Differential electron density images. Reproduced with permission [88]. Copyright 2025, Springer Nature. (g) Overpotential of SACs covering *3d*, *4d*, and *5d* transition metals with different coordination structure. Reproduced with permission [82]. Copyright 2024, Springer Nature. (h) Schematic diagram of Li_2_S_6_‐Fe‐P_4_. Reproduced with permission [93]. Copyright 2025, Wiley‐VCH GmbH.

Different from the strategy of constructing metastable M─N_3_P_1_ coordination environments to induce localized electron polarization and boost intrinsic catalytic activity, introducing extra P atoms to form N_2_P_2_ coordination can further rearrange electron cloud distribution for enhanced catalytic performance [82, 83]. Additional P heteroatoms introduce more intrinsic defects into carbon lattices, generating extensively uneven charge distribution and creating abundant catalytically active sites [84, 85]. Xu et al. fabricated Co─N_2_P_2_ SACs on a carbon layer (CoPNC) and elaborated the structure‐activity relationship among metastable M─N_2_P_2_ SACs configuration, charge transfer behavior, and catalytic conversion kinetics of LiPSs [86]. Compared with Li_2_S_6_ adsorbed on Co─N_4_, electrons in the Li_2_S_6_‐Co─N_2_P_2_ predominantly accumulate between P and Li atoms, with charge dissipation regions spread across the planar Li─S bonding, demonstrating more concentrated electron aggregation. Meanwhile, the S─S bond length in the Li_2_S_6_‐Co─N_2_P_2_ stretches to 2.229 Å, longer than that in Li_2_S_6_‐Co─N_4_ (2.163 Å), which verifies that the evident conformational distortion of Li_2_S_6_ triggered by the Co─N_2_P_2_ coordination configuration accelerates the decomposition of LiPSs. As shown in Figure 4e, the decomposition energy barrier of Li_2_S on CoPNC (1.65 eV) is distinctly lower than those of CoNC (1.72 eV) and PNC (1.81 eV), which was also verified by in‐situ Raman and Li_2_S nucleation tests, solidifying the critical role of asymmetric N_2_P_2_ coordination in optimizing the catalytic performance of SACs [87]. The strong electronegativity of N atoms and the rigid, stable M─N_4_ coordination structure greatly stabilize central metal atoms yet compromise catalytic activity. To address this issue, Ma et al. fabricated metastable single‐atom active sites with Co─N_2_P_2_ coordination and systematically investigated the local electron transfer mechanism [88]. Theoretical simulations in Figure 4f reveal that Co‐P bonds are shorter than Co‐N bonds, which induces an asymmetric geometric configuration and uneven electron distribution in Co─N_2_P_2_. Likewise, P, with lower electronegativity, elevates the density of free electrons on central Co atoms, lowers the Fermi level, and accelerates electron transport [89]. Meanwhile, P‐doping and asymmetric coordination introduce abundant intrinsic defects and enlarge the specific surface area, which facilitate Li^+^ diffusion and exposes more active sites for LiPSs adsorption and conversion [90]. Peng et al. reported that P‐doping effectively reduces electrocatalytic overpotential and explored the structure‐activity relationship between catalytic performance and TM‐C_1_P_3_ and C_2_P_2_ [63, 89]. The DFT results in Figure 4g demonstrate that using 3d transition metals (Fe, Co, Ni) as central metal sites can deliver the lowest overpotential for LiPSs redox kinetics. Among them, Fe‐based SACs exhibit superior charge transfer capacity between Fe centers and LiPSs, with Fe‐C_2_P_2_ achieving the minimum overpotential of only 0.042 V. DFT calculations further prove that TM‐C_2_P_2_ coordination outperforms both the stable TM─N_4_ configuration and metastable M─N_3_Cl structure in catalytic activity, making low‐cost, and highly activity Fe‐C_2_P_2_ for Li─S batteries. Moreover, among *3d*, *4d* and *5d* metal centers, 3d metals serve as optimal single‐atom sites with outstanding catalytic activity toward LiPSs. What's more, owing to the low electronegativity of P, P atoms share the valence electrons with S and rapidly shuttle electrons from the C skeleton to the adsorbed LiPSs [91]. Thus, benefiting from the synergistic effect of M‐S and P─S bonds, the Fe‐C_2_P_2_ achieves strong immobilization of LiPSs and rapid cleavage of S─S bonds, thus boosting the nucleation kinetics of Li_2_S.

P coordination increases the number of free electrons on the central metal atoms, and the synergy between P‐S and TM─S bonds strengthens the anchoring capability toward LiPSs (Figure 4h) [92]. On this theoretical basis, Chen et al. designed SACs with a stable Fe‐P_4_ coordination configuration (FePC) to improve the intrinsic catalytic activity of the central Fe sites [93]. Compared with highly electronegative N atoms, P enables faster charge transport between Fe and the coordinating atoms. Ex‐situ XPS characterizations of FePC before and after Li_2_S_n_ adsorption also verify that Fe and P act as Lewis acid and base sites, respectively, anchoring Li and S species separately and trapping soluble LiPSs during electrochemical cycling. Moreover, the electronic modulation of Fe energy bands by P reduces the reaction energy barrier for the conversion of Li_2_S to Li_2_S_2_ on Fe‐P_4_‐C from 1.43 eV (Fe─N_4_‐C) to 1.27 eV, which confirms that Fe‐P_4_ coordination accelerates the redox kinetics of LiPSs compared with the conventional Fe─N_4_ coordination.

S has an electronegativity of 2.58, comparable to C (χ_c_ = 2.55) yet lower than N (χ_N_ = 3.04), resulting in negligible electron polarization between S and C and enabling continuous electron transport (Figure 5a) [78]. Meanwhile, when S replaces C or N coordination sites, the quantity of local free electrons rises due to the 6 valence electrons of S, which is more than those of C and N, thus generating high spin density and accelerating local charge transfer. In our previous work, substituting O sites with S and tuning the substitution ratio in electrocatalysts led to a volcano‐type trend in catalytic activity [94]. Thus, regulating heteroatom substitution (e.g., replacing N with S) can transform the stable M─N_4_ framework into metastable M─N_4‐x_S_x_ and ultimately stable M‐S_4_ configurations. Accordingly, the electrocatalytic activity is expected to vary with the substitution amount of coordinating heteroatoms. For instance, Zhang et al. adopted SACo as the model, substituted N sites with S atoms, and established the theoretical models of Co─N_3_S_1_, Co─N_2_S_2_ and Co─N_1_S_3_ SACs to explore how heteroatom substitution modulates the electrocatalytic performance of SACs (Figure 5b) [95]. Figure 5c reveals that the Bader charge of Co decreases from ‐0.83 e^−^ (Co─N_4_) to ‐0.64 e^−^ (Co─N_1_S_3_), indicating that the substitution of S reduces the electron density near the Fermi level, which impedes electron migration. Nevertheless, S substitution strengthens orbital hybridization between S 2p and Co 3d orbitals and elevates the local electrostatic potential around Co atoms, synergistically reinforcing the interaction between Co centers and LiPSs. However, excessive S substitution generates overly strong coordination between Co and LiPSs, saturating catalytic active sites and suppressing subsequent redox reactions (Figure 5d). Moreover, asymmetric coordination of Co‐S and Co‐N induces electron polarization. Consequently, the asymmetric Co─N_3_S_1_ coordination configuration delivers reduced charge transfer resistance and superior LiPSs redox kinetics. The specific capacity of Co─N_3_S_1_/SeSPAN‐based Li─S batteries can still retain 99.24% after the 1000 cycles at 1.0 C. Based on the theory above, Li et al. constructed interconnected 3D porous carbon networks with asymmetric V─N_3_S_1_ coordination to realize long‐range ordered electron transport and boost the conversion kinetics of LiPSs [96]. The asymmetric coordination of N and S combined with the large atomic radius of S, induces positional displacement of the central V atoms and reduces the Bader charge (+1.36 e^−^ vs. +1.47 e^−^, Figure 5e), which reflects an increased number of mobile free electrons, shifts the d‐band center of V downward, thus accelerating the catalytic conversion of LiPSs [97]. Specifically, in Li_2_S nucleation process, the V─N_3_S_1_ delivers a higher nucleation current (0.28 mA mg^−1^) and larger nucleation capacity (373.3 mAh g^−1^), compared with the V─N_4_ (0.18 mA mg^−1^, 246.5 mAh g^−1^). SEM images show uniform Li_2_S deposition on carbon paper loaded with V─N_3_S_1_, while severe Li_2_S agglomerates form on V─N_4_, further confirming the superior catalytic performance of V─N_3_S_1_. Moreover, the introduction of coordinating heteroatoms such as P and S induces obvious geometric distortion of the original M─N_4_ geometric configuration, which shifts the d‐band center, modulates the adsorption geometry, bond lengths and bond angles of LiPSs on SACs, and ultimately reduces the energy barriers for LiPSs catalytic conversion (Figure 5f). To further explore the universal applicability of asymmetric N_3_S_1_ coordination in enhancing SACs activity, Ling et al. fabricated asymmetric Fe─N_3_S_1_ SACs and revealed that asymmetric coordination constructed by electron‐rich, weakly electronegative heteroatom substitution of N triggers electron redistribution and generates abundant electrocatalytic active sites [98]. DFT calculations in Figure 5g further verify that replacing N with S shifts the d‐band center of central Fe upward and strengthens hybridization between Fe 3d orbitals and S 2p orbitals of LiPSs, facilitating rapid local charge transfer and effective adsorption of LiPSs. Moreover, the Fe─N_3_S_1_ bridging structure of FeSAC‐NS can anchor Li^+^ with the strong adsorption energy of ‐1.11 eV and accelerate fast desolvation of Li(solvents)_x_^+^, which releases abundant free Li^+^ to expedite LiPSs conversion [99].

**FIGURE 5:**
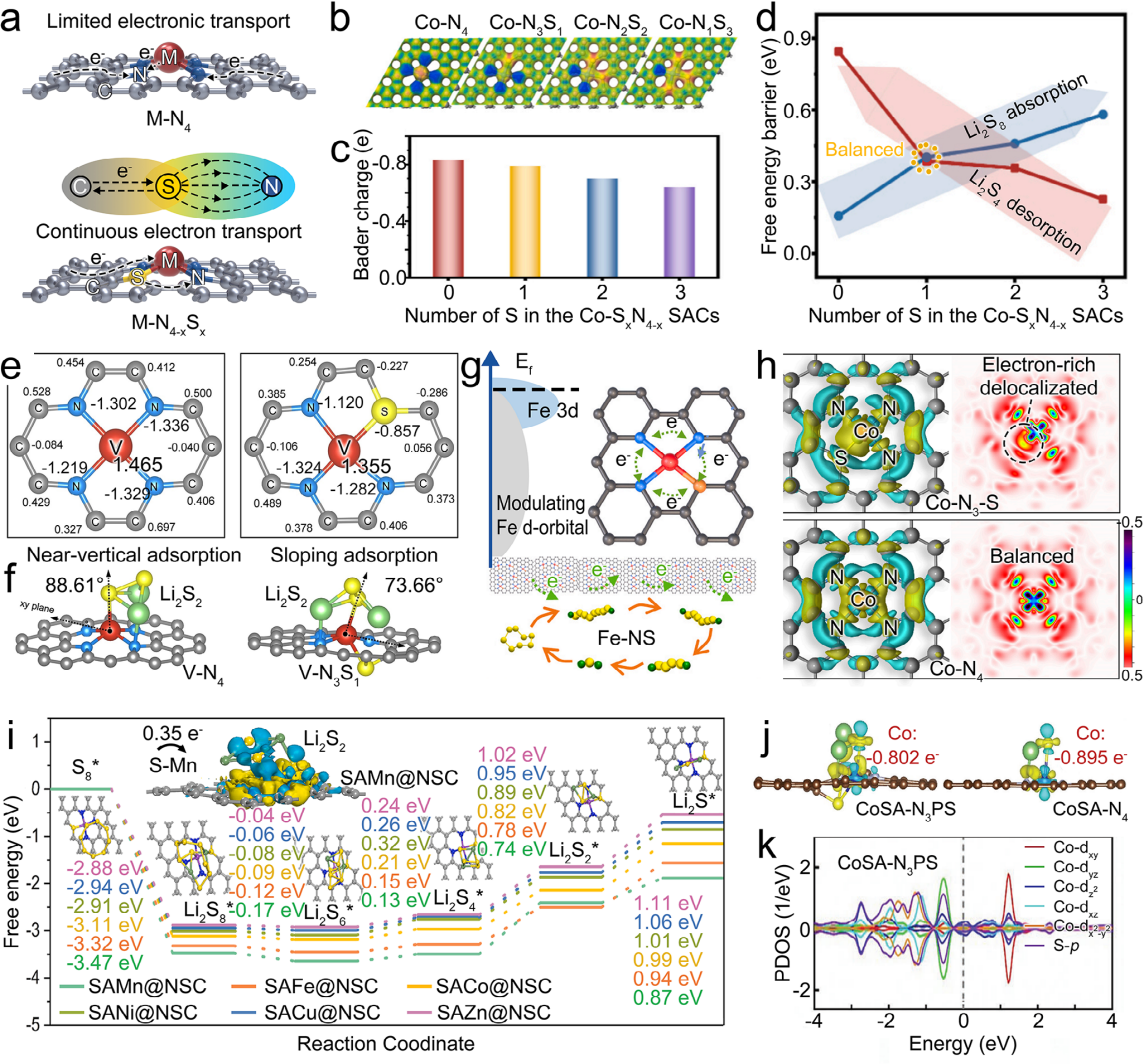
(a) Schematic mechanism of local charge redistribution induced by substituting N with weakly electronegative S heteroatom. (b) Differential electron density image of Co─N_4‐x_S_x_. (c) Bader charge of Co in Co─N_4‐x_S_x_. (d) Free energy barrier of Li_2_S_n_ absorption/desorption on Co─N_4‐x_S_x_. Reproduced with permission [95]. Copyright 2025, Elsevier B.V. (e) Bader charge numbers of atoms in V─N_4_ and V─N_3_S_1_. (f) The adsorption optimized structure of Li_2_S_2_ on the V─N_4_ and V─N_3_S_1_. Reproduced with permission [96]. Copyright 2024, Wiley‐VCH GmbH. (g) Schematic illustration of the working mechanism of FeSAC‐NS. Reproduced with permission [98]. Copyright 2025, Elsevier B.V. (h) Differential charge density of Co─N_4_ and Co─N_3_S. Reproduced with permission [100]. Copyright 2025, American Chemical Society. (i) Gibbs free energy diagrams of sulfur species reduction. Reproduced with permission [101]. Copyright 2024, Elsevier B.V. (j) Differential charge density and (k) PDOS of Co‐d orbitals and S‐p orbitals. Reproduced with permission [102]. Copyright 2024, Wiley‐VCH GmbH.

P, which has the same number of valence electrons as N (5 e^−^) but lower electronegativity, replaces N sites to construct asymmetric Co─N_3_P SACs, inducing localized electron polarization and elevating the catalytic activity of central Co atoms. However, this strategy only produces asymmetric charge distribution without generating extra free electrons. Correspondingly, the decomposition energy barrier of Li_2_S decreases from 1.39 eV on Co─N_4_ to 1.11 eV on Co─N_3_S_1_. Thus, Li et al. reported that the incorporation of electron‐rich S supplies abundant free electrons forto Co─N_3_S_1_, and the low electronegativity of S promotes the delocalization of excess electrons, which creates extensive charge accumulation regions between Co and S atoms and accelerates electron transfer from Co centers to LiPSs (Figure 5h) [100]. What's more, the shift of the d‐band center (‐0.71 eV), the stronger hybridization between Co 3d and S 2p orbitals in the PDOS spectra, and the formation of more antibonding states further validate the above theoretical deductions. Asymmetric M─N_3_S_1_ coordination can modulate the d‐band center of central metal atoms and strengthen orbital hybridization between TMs and S 2p orbitals, thereby improving the LiPSs anchoring capacity and accelerating the cleavage of S‐S and Li─S bonds. To further screen TM─N_3_S_1_ configurations with optimal catalytic activity, Xu et al. investigated the interactions between LiPSs and Mn‐, Fe‐, Co‐, Ni‐, Cu‐, and Zn‐based TM─N_3_S_1_ SACs, and demonstrated that the introduction of TM significantly improves the electronic conductivity of the substrate and enhances charge transfer efficiency [101]. DFT calculations in Figure 5i reveal that Mn─N_3_S_1_ possesses the highest d‐band center, enabling strong anchoring of Li_2_S_6_ (2.97 eV), elongating S─S bonds to 2.93 Å and shortening Mn─S bonds to 2.35 Å, thus accelerating LiPSs conversion (0.87 eV). Differential charge density analysis further confirms that more charge transfers from Li_2_S_2_ to Mn─N_3_S_1_ (0.35 e^−^) than to Mn─N_4_ (0.12 e^−^), strengthening charge transfer between Mn and Li_2_S_2_ and speeding up the conversion kinetics of Li_2_S_2_ to Li_2_S. Consequently, compared with S/NC and S/NSC‐based Li─S batteries, the S/SAMn@NSC‐based cell exhibits an extremely low polarization overpotential of 70.1 mV at 0.1 C.

Introducing a single type of nonmetallic heteroatom into the symmetric M─N_4_ coordination structure breaks structural equilibrium and accelerates local electron transfer. In contrast, incorporating two or more types of nonmetallic heteroatoms leverages the synergistic charge competition effect among heteroatoms to tailor a more favorable electronic configuration for central metal atoms, which strengthens orbital hybridization between the 3d and 2p orbitals. Mai's group fabricated SACs with asymmetric Co─N_3_PS coordination via a two‐step pyrolysis strategy [102]. The amount of charge transferred between Li_2_S and the catalyst shifts from ‐0.895 e^−^ for SACo─N_4_ to ‐0.802 e^−^ for SACo─N_3_PS, indicating that more electrons are retained in the 3d orbitals of central Co atoms (Figure 5j–k). Benefiting from the synergistic effect of P and S coordinating atoms, the binding strength between LiPSs and Co─N_3_PS is greatly enhanced, which effectively improves sulfur utilization. Time‐of‐flight secondary ion mass spectrometry (TOF‐SIMS) characterizations further demonstrate that LiPSs are uniformly confined within the nanocage structure of CoSA─N_3_PS. In comparison, severe agglomeration of LiPSs occurs on conventional S@NPS electrodes, reducing the number of accessible catalytic sites and triggering the shuttling of unanchored LiPSs.

Variations in the charge transfer quantity between coordinating atoms and central metal sites lead to distinct catalytic activity [103, 104]. Unlike the above‐mentioned M─N_3_Cl SAC systems with Cu as the metal center, Yang et al. selected Mn as the central metal to elaborate on the regulatory effect of asymmetric N_3_Cl coordination on the catalytic activity of Mn sites [105]. They constructed Mn─N_3_Cl active sites on N, B co‐doped carbon nanotubes (MnN_3_Cl@BNC) through pyrolysis combined with ultraviolet irradiation purification. Electrochemical and theoretical results reveal that the introduction of Cl reduces the decomposition energy barrier of LiPSs on MnN_3_Cl@BNC to 1.67 eV, much lower than those of symmetric Mn─N_4_ (2.01 eV) and unsaturated Mn─N_3_ coordination (1.86 eV), proving that Cl heteroatom coordination positively boosts catalytic activity. Additionally, Cl incorporation narrows the band gap of Mn─N_3_Cl, further elevating the overall catalytic performance of the material [106]. Accordingly, the MnN_3_Cl@BNC‐based Li─S cell delivers a high discharge specific capacity of 743 mAh g^−1^ even under a high current rate of 3.0 C.

Apart from substituting N with other nonmetallic heteroatoms to construct asymmetric coordination configurations for improved catalytic activity, the targeted screening of nonmetallic heteroatoms with abundant valence electrons and low electronegativity, such as S and P, can supply extra free electrons to the central metal sites, thereby facilitating electron delocalization, expands the radius of electron sharing, and enables rapid trapping and catalytic conversion of LiPSs.

### 2.2 Unsaturated Coordination

Symmetric M─N_4_ coordination leads to weak polarity of the central metal atoms. Disrupting the balanced coordination environment induces electron cloud polarization and endows the central metal sites with stronger polar adsorption capability [107]. Specifically, converting fully saturated M─N_4_ coordination into unsaturated M─N_x_ configurations generates coordination defects, triggers charge redistribution on the central metal atoms, and accelerates the charge transfer kinetics between SACs and LiPSs (Figure 6a) [108, 109].

**FIGURE 6:**
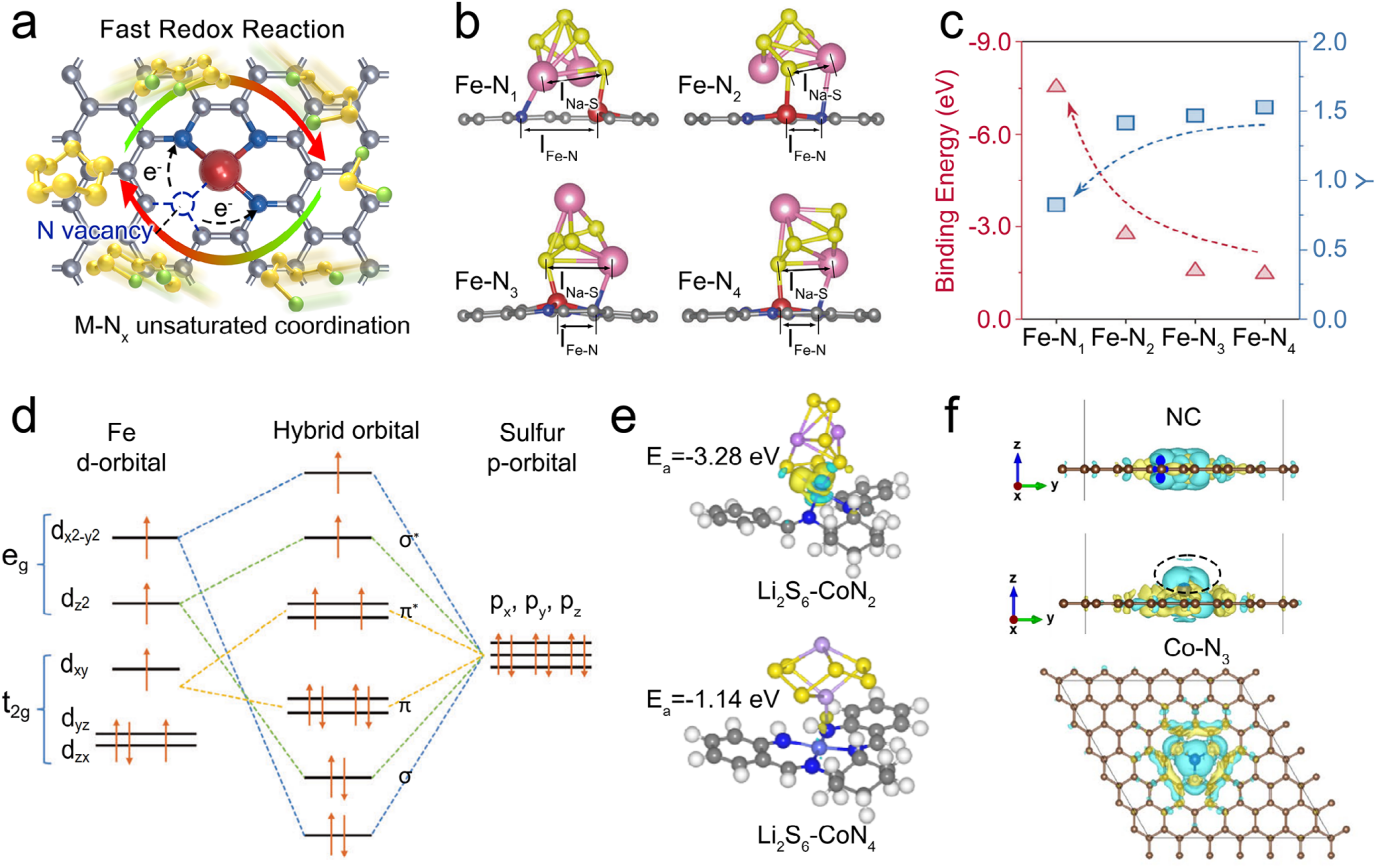
(a) Schematic diagram for boosted LiPSs redox via unsaturated M─N_x_ coordination. (b) Configurations of Na_2_S_4_ adsorbed on Fe─N_x_. (c) relationships among unsaturation degrees of Fe─N_x_, binding energies, and geometric structure descriptors. (d) Schematic for the d‐p orbital hybridization between Fe─N_x_ and sulfur species. Reproduced with permission [110]. Copyright 2025, Springer Nature. (e) Adsorption of Li_2_S_6_‐CoN_2_ and CoN_4_. Reproduced with permission [55]. Copyright 2025, American Chemical Society. (f) Differential charge density of Co─N_3_. Reproduced with permission [111]. Copyright 2025, Springer Nature.

To investigate how M─N_x_ modulates the charge distribution of central metal atoms, Hu et al. synthesized Fe─N_x_ coordination(x = 1–4) by regulating the pyrolysis temperature, demonstrating that reducing the number of coordinated N atoms to construct defective configurations alleviates steric hindrance for S_n_^2−^ adsorption [110]. Meanwhile, this work also defines a parameter γ as the ratio of Na─S bond length to Fe─N bond length, which exhibits an inverse correlation with adsorption energy (Figure 6b). Figure 6c reveals that the γ value of Fe─N_1_ combined with Na_2_S_4_ is 0.824, much lower than the value of 1.527 for Na_2_S_4_‐Fe─N_4_. Correspondingly, the adsorption energy of Na_2_S_4_ decreases from ‐1.467 eV (Fe─N_4_) to ‐7.532 eV (Fe─N_1_). Unsaturated coordination provides additional hybrid orbitals on the central metal atoms to form d‐p orbital hybridization with S, facilitating local charge transfer (Figure 6d). Therefore, highly unsaturated Fe─N_1_ sites boost the decomposition kinetics of sodium polysulfides and lower the corresponding energy barrier. What's more, the unsaturated M─N_x_ coordination can modulate the d‐band center of central metal atoms, reduce the occupation of antibonding orbitals, and strengthen charge transfer between M─N_x_ sites and LiPSs. Geng et al. verified via theoretical calculations and electrochemical measurements that Co─N_2_ coordination greatly strengthens the interaction between Co centers and LiPSs, thereby suppressing the shuttling effect of LiPSs [55]. Specifically, unsaturated Co atoms with low oxidation states exhibit intensified orbital hybridization between Co 3d and S 2p orbitals of LiPSs, which reinforces M─S bonding interactions (Figure 6e). Park et al. immobilized low‐coordinate Co─N_3_ SACs on porous carbon nanofibers (CoSA/p‐CNF) to realize efficient adsorption and catalytic conversion of polar LiPSs [111]. The interconnected porous carbon skeleton enables unobstructed electron transport and exposes abundant SACs active sites, achieving superior electrocatalytic performance. Furthermore, structural optimization indicates that the central Co atom and the three N atoms in Co─N_3_ do not lie coplanar, which allows electrons to accumulate along the z‐axis toward the N atoms, expanding the range of 3D delocalized electron‐sharing, thus realizing LiPSs‐capture and reducing the energy barrier (1.22 eV) for the conversion of Li_2_S_4_ to Li_2_S on Co─N_3_ (Figure 6f). In addition, asymmetric low‐coordinate Co─N_3_ sites strengthen Co‐S and Li‐N bonds, raising the adsorption energy of Li_2_S_6_ from 1.33 eV (Co─N_4_) to 2.37 eV (Co─N_3_) [112]. Specifically, the trigonal pyramidal spatial geometry of Co─N_3_ distorts adsorbed Li_2_S_6_ molecules and accelerates the cleavage of S─S bonds. Consequently, the discharge capacity of Li─S pouch cells equipped with CoSA/p─CNF interlayers retains 92.2% after 150 cycles at 0.5 C.

Substituting carbon atoms adjacent to SACs with heteroatoms such as O, N and S disrupts the periodic charge distribution on the substrate surface and triggers redistribution of local electric fields. In particular, the incorporation of electron‐rich S with low electronegativity drives electron polarization toward coordinated N sites, broadening the electron‐sharing region of M─N_x_ SAC centers. Gao et al. immobilized unsaturated Ni─N_3_ SACs on N, S co‐doped porous carbon and verified that unsaturated coordination and heteroatom doping jointly strengthen the interaction between Ni and LiPSs, endowing the Ni‐NSC‐based Li─S cell with outstanding cycling stability (4.7 mAh cm^−2^ after 100 cycles at 0.2 C) [113]. Computational results reveal that co‐doping N and S heteroatoms increases the charge transfer magnitude between central Ni and Li_2_S_4_ from ‐0.649 e^−^(Ni─N_3_‐C) to ‐0.808 e^−^(Ni─N_3_‐NSC), elevating the adsorption energy toward Li_2_S_4_ to ‐1.55 eV and enabling efficient immobilization of LiPSs. Meanwhile, compared with Ni─N_4_ coordination, the Ni‐N bond length in Ni─N_3_ shrinks to 1.85 Å, shifting electron clouds closer to N atoms, which facilitates electron loss from central Ni sites and further strengthens the adsorption affinity of Ni toward LiPSs.

Thus, broking the symmetric coordination alters the ionic‐covalent mixing ratio of M‐coordination atom bonds, adjusting orbital matching degree and bond order, and further modulating the Lewis acidity/basicity of central metal atoms. Besides, the optimized Lewis acid‐base interaction effectively regulates the affinity toward LiPSs, balances the LiPSs adsorption strength and redox reversibility, and ultimately promotes efficient LiPSs conversion.

### 2.3 Axial Coordination

As demonstrated in the above investigations, the expanded range of electron sharing along the z‐axis strengthens the interaction with LiPSs and accelerates charge transfer between metal and sulfur species. Accordingly, beyond engineering planar M─N_4_ coordination structure, rational optimization of the orientation and spatial position of coordinating atoms in 3D space can induce lattice distortion and enhance SAC activity (Figure 7a). Qu et al. adopted CuO as the substrate and adopted the covalent axial coordination strategy to incorporate O atoms into the Fe─N_4_ coordination environment (FePc@CuO), which narrows the d‐band center gap of Fe from 1.71 to 1.31 eV and facilitates charge transfer between Fe and LiPSs (0.147 e^−^, Figure 7b) [114]. Meanwhile, the axial O coordination transforms the spin state of Fe from intermediate spin (IS, d_xz_^2^d_yz_^2^d_z_^21^d_xy_^1^) to low spin (LS, d_xy_^2^d_xz_^2^dy_z_^2^), intensifying d‐p orbital hybridization between Fe and S, promoting the delocalization of free electrons within relevant orbitals, and further improving local charge transfer efficiency. Moreover, DFT calculations further reveal that the Fe 3d orbitals in FePc@CuO undergo energy level rearrangement and electron delocalization. Obvious charge transfer occurs along the Fe‐O‐Cu bridge, accompanied by a remarkable reduction in the magnetic moment of Fe atoms, which accelerates the catalytic conversion of LiPSs intermediates. As shown in Figure 7c, in‐situ Raman characterizations further confirm that axial O coordination endows Fe─N bonds with higher structural flexibility, enabling them to actively participate in the redox conversion of LiPSs. In contrast, the Raman signals of Fe─N bonds in pristine Fe─N_4_ SACs remain unchanged throughout the reaction. Furthermore, the axial coordination strategy is extended to SAMn, SACo, SANi, all of which exhibited analogous structural evolution trends. Thus, axial coordination modification effectively elevates the intrinsic catalytic activity of central metal sites and improves the overall catalytic performance of SACs.

**FIGURE 7:**
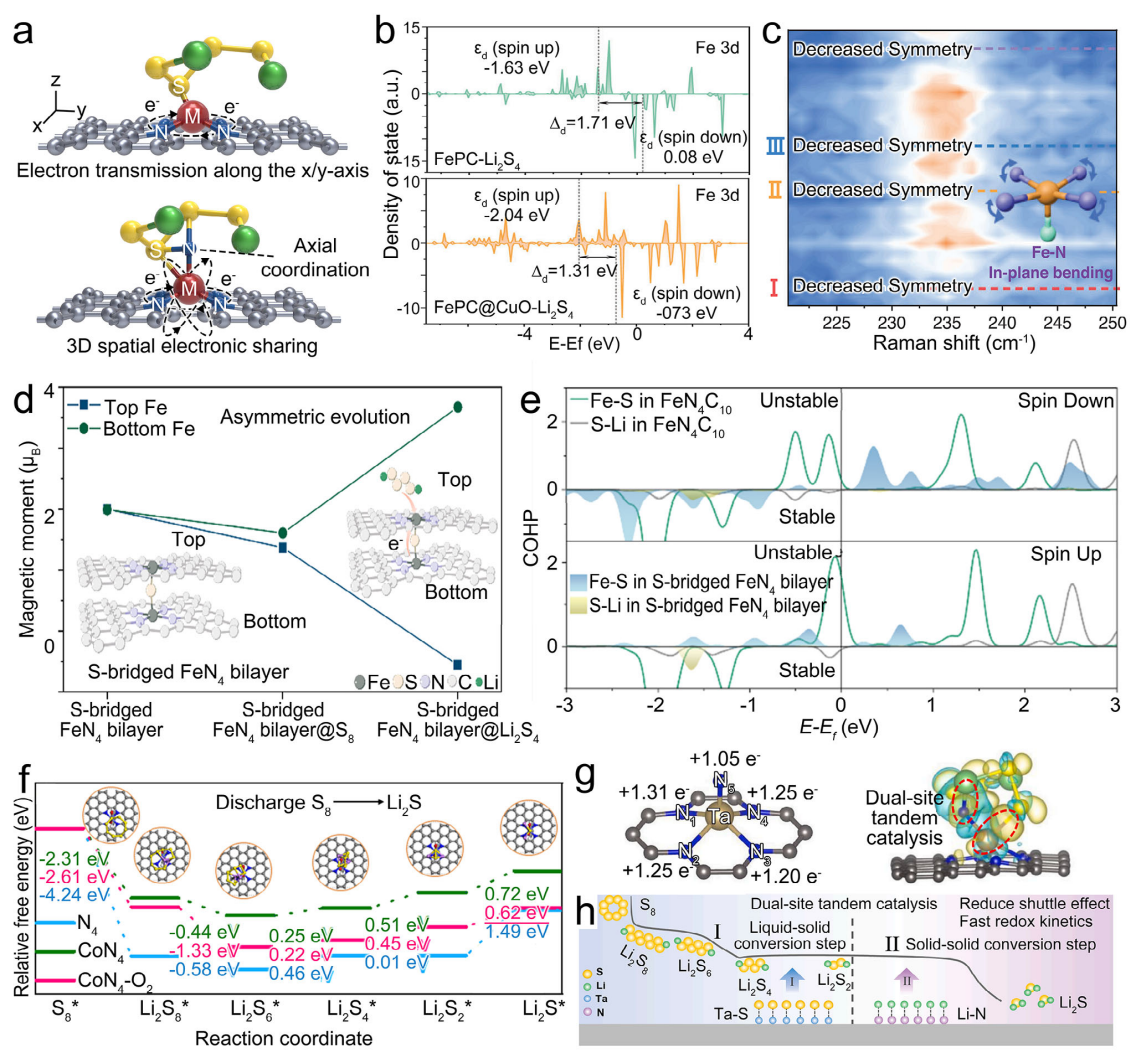
(a) Schematic illustration of axial coordination atoms inducing local electron redistribution. (b) Density of state. (c) In‐situ Raman. Reproduced with permission [114]. Copyright 2024, Wiley‐VCH GmbH. (d) Comparison of magnetic moments of the top and bottom Fe atoms under different conditions. (e) COHP analysis of Fe‐S and Li─S bonds in the S‐bridged FeN_4_ bilayer and FeN_4_C_10_ structures. Reproduced with permission [116]. Copyright 2025, American Chemical Society. (f) Calculated free energy diagram. Reproduced with permission [117]. Copyright 2025, American Chemical Society. (g) Bader charge numbers of coordination atoms of Ta─N_5_. (h) Schematic illustration of the mechanism of dual‐site Ta─N_5_ catalysis. Reproduced with permission [119]. Copyright 2025, Elsevier B.V.

The catalytic activity of SACs primarily originates from the degree of d‐p orbital hybridization between central metal sites and LiPSs. Constructing asymmetric coordination, tuning energy levels to induce electron cloud polarization, facilitating electron delocalization, and modulating spin states can adjust the d‐orbital parameters of central metals, strengthen d‐p orbital hybridization, and accelerate local electron transfer [115]. Among various regulation strategies, axially coordinated atoms with specific spatial orientations enable the d_xz_ and d_yz_ orbitals of TMs to readily form π‐type interactions with LiPSs, realizing efficient electron sharing. Cabot et al. bridged two layers of Fe─N_4_ active sites via axially coordinated S atoms (Fe‐SNC), which promoted electron delocalization within the 3d orbitals of central Fe atoms, achieved dynamic spatial electron transfer and establishes the 3D electron transport network (Figure 7d) [116]. PDOS analysis reveals that axial S bridging induces prominent electron delocalization in the d_yz_, d_xz_ and d_z_^2^ orbitals of Fe, and enlarges the orbital overlap region between S 2p and Fe 3d orbitals, indicating rapid axial electron migration. During electrochemical reactions, the top and bottom Fe sites regulate charge distribution through the central bridging S atom to sustain continuous electron supply. More importantly, the S bridge shifts the COHP value of Fe‐SNC from negative to nearly zero, which confirms strengthened Fe‐S interactions and substantially weakened Li─S bonding, lowering the decomposition energy barrier of Li_2_S to 1.14 eV (Figure 7e). Further investigations demonstrate that dual Fe─S bonds and Li‐N bonds endow Fe‐SNC with the strong Li_2_S_4_ adsorption energy (‐1.62 eV) for rapid LiPSs capture, which was validated by Li_2_S_4_ adsorption experiments and in‐situ Raman characterizations. Axial coordination not only creates an asymmetric electronic coordination environment to reduce the energy barrier of LiPSs conversion, but also enables dual‐metal‐site catalysis via bridging coordinating atoms. Yan et al. constructed single‐atom active sites with Co─N_4_─O_2_ coordination through axial O coordination [117]. The synergistic effect between top and bottom Co atoms facilitates complete dissociation and oxidation of Li_2_S and prevents the coverage of catalytic active sites. Meanwhile, axial O coordination elevates the valence state of Co from +2.0 (Co─N_4_) to +2.52 (Co─N_4_─O_2_), slightly reducing the adsorption affinity toward LiPSs. Nevertheless, axial O coordination shifts the density of states of Co d_z_^2^ and d_x_^2^_‐y_^2^ orbitals closer to the Fermi level, enhancing the intrinsic catalytic activity of Co. Consequently, the Co─N_4_─O_2_ configuration delivers the lowest energy barrier (0.62 eV) for the conversion of Li_2_S_2_ to Li_2_S (Figure 7f). Therefore, the Co─N_4_─O_2_@CMK‐5 based Li─S cell exhibits nearly negligible capacity decay over 500 cycles even at 5.0 C. What's more, Zhou et al. fabricated Ti─O_5_ based SACs on reduced graphene oxide (rGO) and explored the effects of M─O_5_ coordination configuration on overall electron distribution and geometric structure [118]. Theoretical calculations reveal that pure rGO possesses a band gap of 0.3 eV and exhibits semiconductor characteristics, attributed to abundant surface defects that impede electron transport. After immobilizing Ti─O_5_, the Fermi level shifts toward the valence band, significantly improving the overall electrical conductivity of the composite. Differential charge density analysis confirms that Ti─O_5_ coordination induces charge accumulation and accelerates charge transfer within Li‐O and Ti─S bonds. In addition, the Li diffusion barrier and Gibbs free energy for LiPSs conversion on Ti─O_5_‐based rGO are markedly reduced, thus accelerating the redox kinetics of LiPSs.

To boost the catalytic activity of 5d‐TM‐based SACs, Sun et al. introduced axially coordinated nitrogen atoms on the planar Ta─N_4_ coordination structure to construct asymmetric Ta─N_5_ coordination [119]. Axial N coordination generates unequal Ta─N bond lengths and inhomogeneous electron distribution (corresponding energy values of 1.25, 1.20, 1.25 and 1.05 eV), which facilitates extensive electron delocalization and sharing (Figure 7g). Meanwhile, the asymmetric coordination induces structural distortion and creates substantial orbital overlap between Ta d_z_^2^ and S p_z_ orbitals, strengthening d‐p orbital hybridization between Ta and S species and elevating catalytic activity. For the Li_2_S_n_‐Ta─N_5_ configuration, the antibonding states of Ta─S bonds solely emerge in the conduction band, which indicates that the electron occupation of antibonding orbitals reinforces the adsorption capacity toward LiPSs. In addition, all bonding states lie below the Fermi level, which accelerates the conversion of Li_2_S_4_ (liquid) into Li_2_S_2_ (solid). Moreover, the 3p orbital electrons of S atoms anchored at Ta sites are activated in the Li_2_S_4_─Ta─N_5_ system, markedly reducing the S─S bond energy and further promoting the conversion of Li_2_S_4_ intermediates (Figure 7h). Ex‐situ XPS characterizations confirm that Li─N coordination and Ta─S bonds gradually grow in quantity during the transformation from Li_2_S_4_ to Li_2_S_2_. When discharging proceeds to 1.70 V with massive Li_2_S generation, the characteristic peaks of Ta─S bonds gradually weaken, demonstrating that Ta─S bonds deliver synergistic catalytic effects in the conversion of Li_2_S_2_ to Li_2_S.

Generally, conventional catalytic conversion of LiPSs proceeds on isolated single‐atom active sites. In contrast, axial coordination enables simultaneous adsorption and catalysis of LiPSs by multiple spatially layered single‐atom sites, which is expected to drastically boost the conversion kinetics of LiPSs.

### 2.4 Dual‐Atoms Catalysts Design

The high solubility of long‐chain LiPSs constitutes the primary origin of the shuttling effect, which contain multiple S atoms, requiring abundant metal sites to form M─S bonds for efficient anchoring [120]. Nevertheless, atomically dispersed SACs fail to deliver synergistic anchoring and catalytic conversion of LiPSs, resulting in continuous capacity decay and poor cycling stability of Li─S batteries [121]. Homonuclear dual‐atom catalysts (DACs) and heteronuclear DACs have recently emerged as promising candidates to improve the sulfur utilization. By integrating two or even three metal atoms within one active site, these catalysts can achieve synergistic adsorption and catalytic transformation of LiPSs. Li et al. revealed through theoretical calculations that homonuclear DACs exhibit symmetric orbital distribution, which enhances orbital overlap and hybridization, strengthens electron delocalization, and accelerates interfacial charge transfer kinetics [122]. Meanwhile, orbital coupling between adjacent metal centers of homonuclear DACs breaks the insulating character of LiPSs intermediates and thermodynamically facilitates the nucleation kinetics of Li_2_S (Figure 8a,b). In contrast, heteronuclear DACs feature asymmetric orbital arrangements, and the different electronegativities drive polarized charge accumulation on one metal center, which cannot convert adsorbed insulating LiPSs into conductive states (Figure 8c). Further theoretical results demonstrate that excessively strong charge transfer between M atoms and S reduces the electron occupation of antibonding orbitals, weakening the adsorption affinity toward LiPSs (Figure 8d). What's more, the correlation between the charge transfer rate of M─S bonds and the conversion energy barrier of Li_2_S_2_ follows a volcano‐type trend, contradicting the conventional view that faster charge transfer always corresponds to a lower Li_2_S_2_ conversion barrier.

**FIGURE 8:**
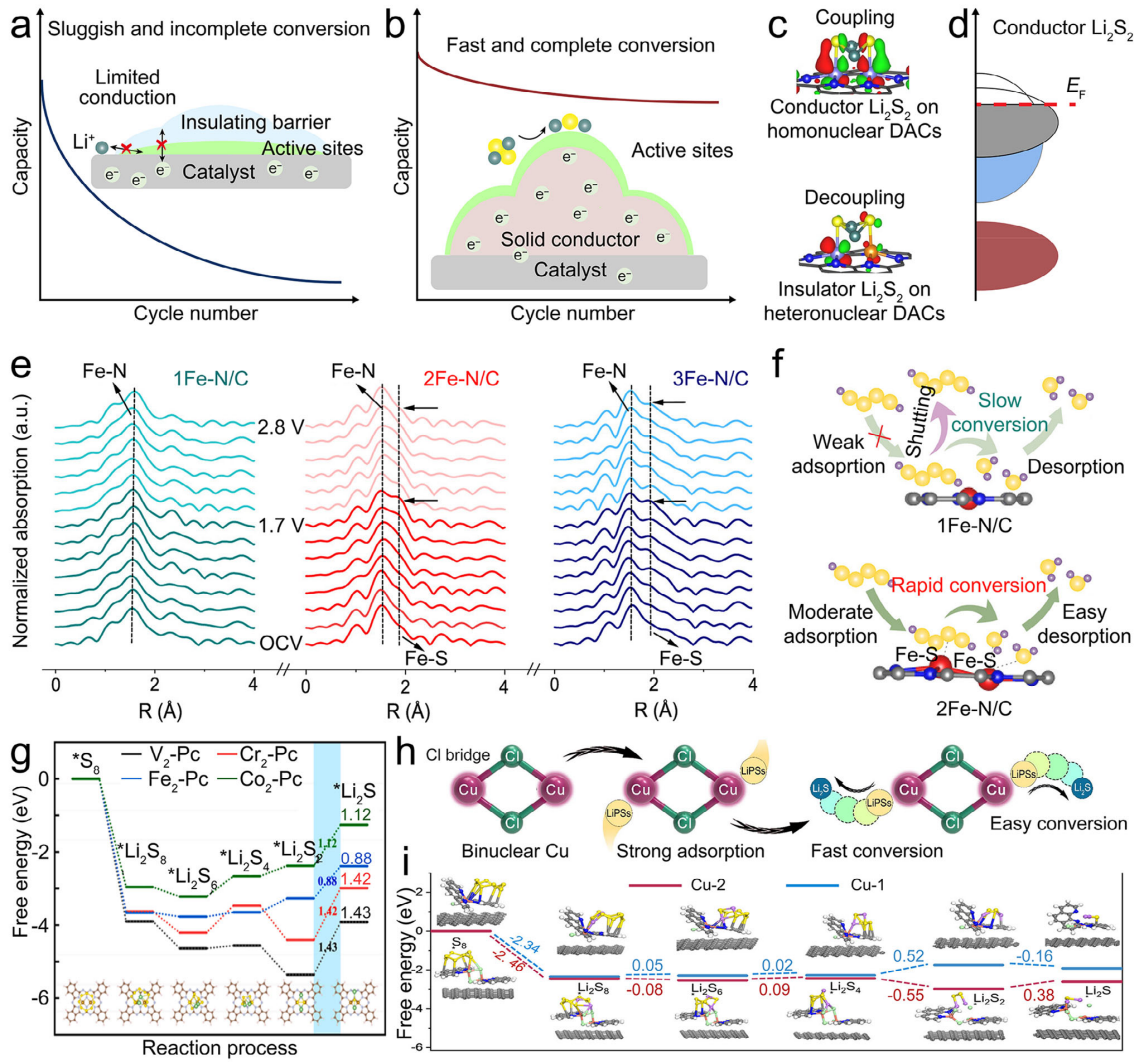
(a) Problems and (b) potential solution for solid‐phase conversion reactions. (c) Comparison of the coupling of the homo‐ and heteronuclear DACs. (d) Influence of orbital hybridization between conductor Li_2_S_2_ and DACs. Reproduced with permission [124]. Copyright 2026, Springer Nature. (e) Operando FTEXAFS spectra of the Fe K‐edge recorded. (f) Schematic of the SRR process on Fe‐based catalysts. Reproduced with permission [123]. Copyright 2024, American Chemical Society. (g) Calculated free energy diagram. Reproduced with permission [125]. Copyright 2025, Elsevier B.V. (h) LiPSs conversions catalyzed by Cu‐1 and Cu‐2. (i) Calculated free energy diagram. Reproduced with permission [127]. Copyright 2024, Springer Nature.

#### 2.4.1 Homonuclear Dual‐Atom Catalysts

Ji et al. discovered a volcano‐type correlation between the catalytic activity toward LiPSs and the number of central metal atoms in SACs, including 1Fe‐N/C, 2Fe‐N/C and 3Fe‐N/C [123]. In 1Fe‐N/C‐based Li─S battery, the Fe─N bonds can be detected during LiPSs conversion catalysis, without obvious Fe‐S signals, which indicates weak interaction between 1Fe centers and LiPSs (Figure 8e). In contrast, in the 3Fe‐N/C‐based Li─S battery, Fe‐S and Fe─N signals could be detected during discharge, yet the Fe‐S signals remained intense throughout the charging process, which demonstrates that excessive adsorption passivates the 3Fe‐N/C active sites, hindering the desorption and catalytic conversion of LiPSs. However, the 2Fe‐N/C‐based cell exhibits gradually intensified Fe‐S signals accompanied by weakened Fe─N bonds during discharge. During Li_2_S oxidation, the Fe‐S peak intensity fades progressively, confirming that 2Fe sites synergistically anchor LiPSs and support the steady catalytic conversion. Moreover, during the reduction process, the valence state of Fe in 2Fe‐N/C declines from +2.48 to +1.95, and fully recovers to its original value after Li_2_S is oxidized back to S_8_, which further verifies that dual Fe sites realize synergistic LiPSs adsorption and rapid charge transfer (Figure 8f). Besides, in 3Fe‐N/C‐based SACs, overly strong adsorption locks the Fe valence state at +1.65, proving that polymetallic sites tend to form excessively strong M─S bonds, impeding LiPSs desorption and elevating the LiPSs conversion energy barrier (1.39 eV). The 2Fe─N_6_ coordination structure achieves a dynamic balance between rapid LiPSs adsorption/dissociation and accelerated S─S bond cleavage, delivering the lowest LiPSs conversion barrier (1.21 eV). Based on DFT calculations, Li et al. screened transition metals, including V, Cr, Fe and Co, and confirmed that Fe‐Fe coordination possesses superior structural stability, narrower band gap, a low Li_2_S nucleation barrier (0.88 eV) and a low Li_2_S decomposition barrier (0.72 eV), all of which collectively boost the electrochemical performance of Li─S batteries (Figure 8g) [124, 125]. Meanwhile, Co‐Co DACs exhibit orbital hybridization characteristics analogous to those of Fe‐Fe species, which balance charge transfer efficiency and M‐N bond stability to improve cycling stability. Nevertheless, Ni‐Ni DACs suffer from weak orbital hybridization and inferior catalytic activity. V‐V and Cr‐Cr DACs bind S intermediates too strongly, leading to blocked active sites and sluggish charge transfer, with high LiPSs conversion barriers (1.43 and 1.42 eV, respectively). As a result, Fe‐Fe DAC‐based Li─S cell retains a discharge specific capacity of 555.8 mAh g^−1^ after 400 cycles at 1.0 C. Thus, the synergistic effect of dual metal centers reorganizes charge distribution and eliminates low‐charge‐density regions to realize high‐efficiency electrocatalysis. Gao et al. fabricated V‐V DACs that deliver strong adsorption toward Li_2_S_6_ (−0.82 eV), and dynamic charge transfer pathways drastically accelerate LiPSs redox kinetics with a low energy barrier (0.09 eV) [126].

It should be noted that although dual‐atom active sites synergistically accelerate LiPSs conversion, conventional N_4_ coordination frameworks often sacrifice catalytic performance to enhance structural stability. To address this issue, Song et al. constructed 2Cu─N_4_Cl_6_ DACs by bridging two Cu─N_2_ moieties via Cl atoms, strengthening the adsorption capacity for Li_2_S_6_ (1.07 eV) and Li_2_S (3.04 eV), which was further validated by in‐situ Raman spectroscopy (Figure 8h‐i) [127]. In addition, dynamic charge modulation through the Cu‐Cl‐Cu bridge gradually reduces the Gibbs free energy for LiPSs conversion on 2Cu─N_4_Cl_6_ to 0.38 eV, enabling uniform electrodeposition of Li_2_S. Accordingly, 2Cu─N_4_Cl_6_ based Li─S pouch cell with a high sulfur loading of 7.7 mg cm^−2^ delivers an ultrahigh initial areal discharge capacity of 7.8 mAh cm^−2^ at 0.1 C.

#### 2.4.2 Heteronuclear Dual‐Atoms Catalysts

Long‐chain and short‐chain LiPSs exhibit distinct metal affinities and anchoring behaviors. Accordingly, it is necessary to introduce multiple metal atomic sites to realize synergistic adsorption and catalysis, accelerate LiPSs conversion, and attain satisfactory electrochemical performance in Li─S batteries [128, 129]. In previous research, the unsaturated 3d orbitals of Fe, Co and Ni together with the high density of states near the Fermi level facilitate rapid anchoring of LiPSs and fast charge transfer across M─S bonds. In particular, Fe features low cost and strong affinity toward sulfur species, yet its intrinsically weak catalytic activity limits the performance improvement of Li─S batteries [130]. To screen DACs with superior sulfur‐philicity and reveal the catalytic mechanisms, Li et al. constructed 15 types of heteronuclear DACs (M_1_M_2_@NG) supported on NG and systematically investigated their electrocatalytic kinetics for LiPSs redox conversion [131]. The NG substrate possesses favorable coordination capacity and structural stability, thus the binding energy of supported metal atoms on NG remains negative, endowing the composite with outstanding thermal stability. Notably, prominent *d‐p* orbital hybridization forms between transition metals and coordinated nitrogen atoms, generating strong electronic coupling and further confirming the structural robustness of M_1_M_2_@NG. Extensive literature has proved that a reaction energy barrier exceeding 1.5 eV thermodynamically inhibits forward sulfur redox reactions. Theoretical simulations demonstrate that dual‐atom catalysts based on V, Fe, Cu, Zr and Pt effectively accelerate LiPSs conversion kinetics. Among them, CuFe@N_6_ delivers low energy barriers of the reduction of Li_2_S_4_ to Li_2_S_2_ (0.65 eV) and for Li_2_S decomposition (0.97 eV), whereas NbFe@N_6_ displays an excessively high barrier (1.99 eV) that suppresses the forward reaction. Meanwhile, the low decomposition barrier of Li_2_S (0.97 eV) on CuFe@N_6_ during Li_2_S oxidation further verifies the outstanding activity of DACs. What's more, CuFe@N_6_ exhibits a moderate adsorption energy toward LiPSs, which simultaneously suppresses the LiPSs shuttle effect and prevents active site poisoning caused by overstrong adsorption. In contrast, FeV@N_6_ and PtZr@N_6_ possess relatively low Li_2_S decomposition barriers (0.92 and 0.95 eV, respectively). However, their excessive adsorption energy (1.31 eV) and high Li_2_S_2_ conversion barriers (1.11 eV) hinder the reduction of LiPSs, resulting in sluggish reaction kinetics in practical applications [132]. The orbital electronic structure of the Li_2_S‐FeCu@N_6_ was further analyzed to uncover the catalytic mechanism of LiPSs on FeCu@N_6_. As shown in Figure 9a, Fe 3d orbitals hybridize with S 3p orbitals to form stable Fe─S bonds for efficient LiPSs adsorption. Meanwhile, Cu 3d orbitals supply abundant antibonding states to activate Li─S bonds and accelerate the cleavage of both Li─S and S─S bonds. Consequently, Fe sites act as anchoring centers for LiPSs intermediates, while Cu sites facilitate the redox transformation of anchored sulfur species; the synergy between Fe and Cu jointly boosts the overall conversion kinetics of LiPSs [133].

**FIGURE 9:**
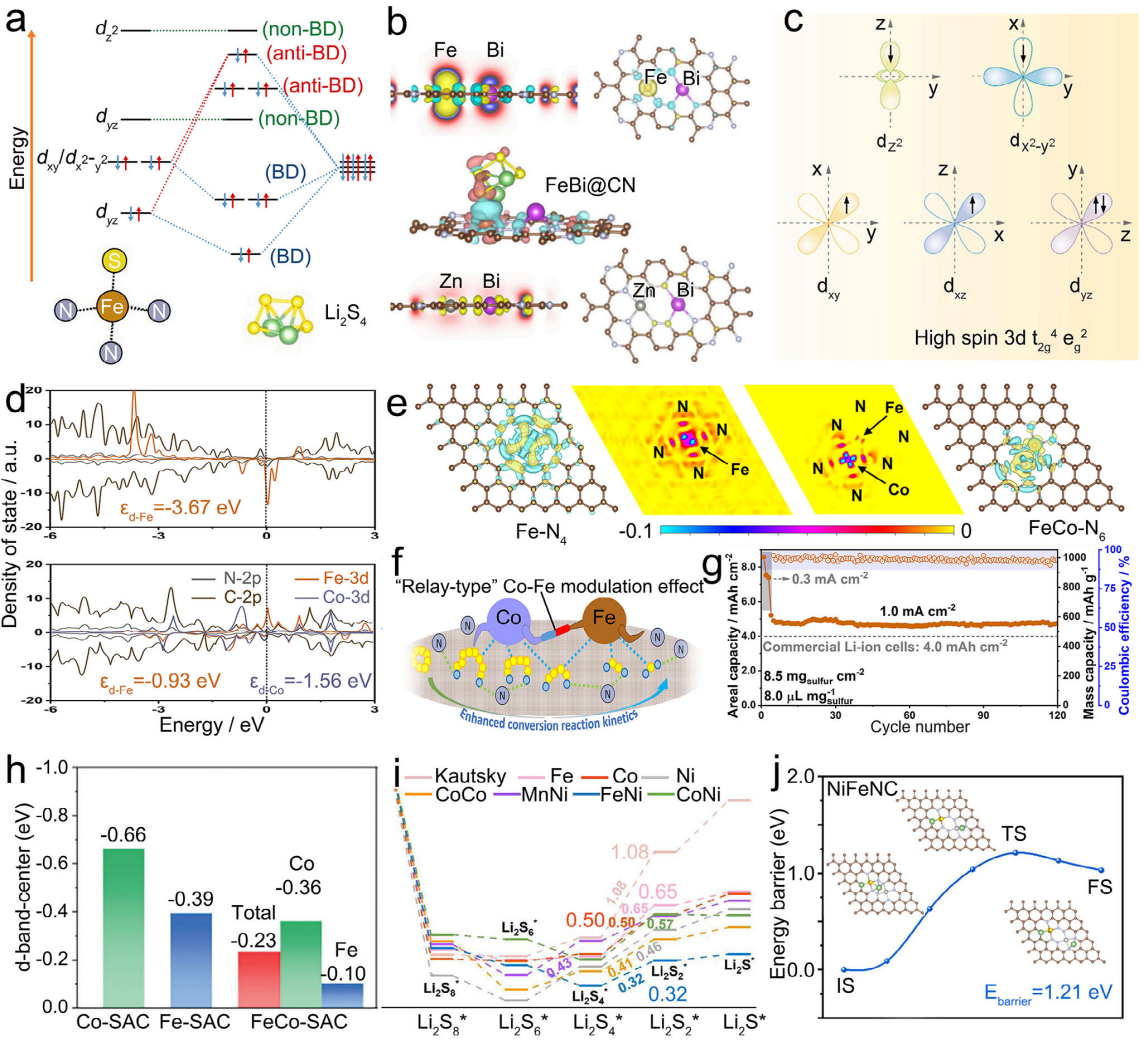
(a) The orbital interaction between Fe and S atoms in the Li_2_S_4_‐Cu‐Fe DACs. Reproduced with permission [122]. Copyright 2026, Elsevier B.V. (b) Charge density difference plots of FeBi@CN and ZnBi@CN. Reproduced with permission [135]. Copyright 2025, American Chemical Society. (c) Spatial illustration of the five 3d orbitals for Fe atoms in high spin state. Reproduced with permission [138]. Copyright 2025, Elsevier B.V. (d) Density of states analysis and the corresponding d‐band center values. (e) Charge density difference and corresponding 2D slices. (f) Mechanism of LiPSs conversion reaction kinetics enhanced by the Co‐Fe DACs. (g) Cycling performance of Co‐Fe DACs‐based Li─S pouch cell. Reproduced with permission [131]. Copyright 2024, American Chemical Society. (h) D‐band center. Reproduced with permission [141]. Copyright 2024, Wiley‐VCH GmbH. (i) Calculated free energy diagram. Reproduced with permission [144]. Copyright 2025, Wiley‐VCH GmbH. (j) Energy evolution during the Li_2_S decomposition process on Ni‐Fe DACs. Reproduced with permission [145]. Copyright 2025, Elsevier B.V.

DACs maintain high atomic utilization efficiency, while leveraging the synergistic interaction between adjacent metal atoms to induce electron cloud polarization and strengthen electronic coupling, thereby delivering superior electrocatalytic activity [134]. Arbiol et al. discovered that although bismuth (Bi, 6s^2^6p^3^) lacks d orbitals, the highly polarized 6p orbitals can form p‐p orbital coupling with S 3p orbitals to realize electron delocalization, reducing the S─S bond energy and lowering reaction barriers [135]. Subsequently, the Fe─Bi─N_4_, with partially filled and highly active Fe (3d^6^), and Zn─Bi─N_4_, with fully filled and low‐activity Zn (3d^10^) DACs were designed to verify the above theory (Figure 9b). DFT calculations reveal that the partially filled 3d orbitals of Fe facilitate electron transfer yet are restricted by the charge transfer rate of LiPSs, limiting the catalytic performance. The Bi atom reconstructs the local electronic structures of Fe and Zn and elevates the spin states of coordinated metal atoms and accelerates local charge transfer, resulting in the band gaps of Fe‐Bi@CN and Zn‐Bi@CN DACs narrowing to 0.25 and 0.48 eV, respectively [136]. More importantly, Bi atom modulates the Fe 3d orbitals and shifts the bonding states of Fe‐S toward the Fermi level, which accelerates the cleavage of S─S bonds. The Gibbs free energy for the conversion of Li_2_S_4_ to Li_2_S on Fe─Bi@CN and Zn─Bi@CN reaches 1.68 and 1.66 eV, respectively, far lower than that on bare CN (2.26 eV).

In SACs, strong interactions between weakly electronegative metal atoms and highly electronegative N atoms weaken the Lewis acidity of metal centers and reduce M─S binding energy. Therefore, multiple metal sites are required to generate synergistic adsorption effects for the efficient capture, adsorption and catalytic conversion of free LiPSs [137]. In addition, polymetallic sites cooperatively regulate the orbital electron occupation of metal atoms and enable ultrafast electron transport. Chen et al. demonstrated that the presence of Co triggers strong spin polarization of Fe 3d orbitals, generates abundant unpaired electrons, promotes electron delocalization and reduces the LiPSs conversion barrier from 0.70 to 0.49 eV [138]. Specifically, Co atom breaks the stable Fe─N_4_ coordination framework and induces a high‐spin configuration for Fe 3d orbitals in the N_3_‐Co‐Fe─N_3_ structure, enabling intensified charge transfer with LiPSs (Figure 9c). When anchored on carbon molecular sieves (CMS), the catalyst achieves strong adsorption of LiPSs with a binding energy of ‐1.94 eV. Electrochemical measurements confirm that benefiting from the synergistic electronic regulation of Co and Fe, symmetric cells assembled with Li_2_S_6_‐Co/Fe‐CMS electrodes exhibit distinct redox responses at a low voltage (0.21 V).

In previous work, we proposed FeCo‐based heterostructures for synergistic LiPSs adsorption and catalysis based on the principle that Fe favors LiPSs capture while Co accelerates the catalytic conversion of LiPSs [130]. Inspired by this strategy, Kim et al. immobilized Fe─Co dual single atoms on MOF‐derived carbon and boosted LiPSs conversion kinetics via electronic coupling between heteronuclear dual atoms [139, 140]. The synergistic adsorption and catalysis of Fe─Co dual atoms reduce electrochemical polarization during LiPSs redox and improve LiPSs conversion efficiency. Although the M─N_4_ coordination shifts the energy bands of monometallic single atoms toward the Fermi level, a noticeable band gap remains between the valence and conduction bands (Figure 9d). In contrast, the synergistic effect of Fe─Co bimetallic sites lifts the d‐band center of Fe─Co DACs and decreases orbital electron occupation, strengthening LiPSs anchoring on Fe─Co DACs, as reported by Li et al. Moreover, the charge transfer from Fe to Co facilitates electron delocalization and enhances the intrinsic electrocatalytic activity of Co sites (Figure 9e) [131]. DFT calculations verify that Li─N, Fe─S and Co─S bonds form in the Li_2_S_n_─Fe─Co─N_6_ coordination structure to immobilize LiPSs intermediates. Intermetallic charge transfer between Fe and Co reinforces Co─S bonds and suppresses the shuttling of LiPSs, a conclusion validated by Li_2_S_n_ adsorption experiments (Figure 9f). Furthermore, Li_2_S nucleation tests and Gibbs free energy calculations confirm that Co accelerates the conversion of Li_2_S_6_ to Li_2_S_4_, whereas Fe promotes the oxidation of Li_2_S_2_. Benefiting from the Fe─Co dual‐atom synergy, the Fe─Co DACs‐based pouch cell retains an areal capacity of 4.8 mAh cm^−2^ after 120 cycles at a current density of 1.0 mA cm^−2^ (Figure 9g). Apparent charge transfer occurs from Fe to Co to form the electron‐rich Co centers within Fe─Co DACs, facilitating charge exchange with LiPSs and lowering catalytic barriers, while the electron‐deficient Fe atoms accommodate S 2p orbital electrons via abundant empty 3d orbitals, strengthening Fe─S bonding energy. Wang et al. reported that Co in Fe─Co DACs possesses an intrinsically narrow band gap, which further narrows the band gap of Fe and triggers Fe 3d orbital splitting to form high‐spin states (Figure 9h) [141]. Differential charge density analysis reveals stronger charge transfer between Li_2_S_6_ and Fe─Co DACs compared with Fe SACs and Co SACs, accelerating the cleavage of Li─S and S─S bonds and delivering an ultralow Li_2_S_2_ conversion barrier (0.19 eV). Meanwhile, the high Li^+^ diffusion barriers on Fe SACs (0.677 eV) and Co SACs (0.661 eV) are also identified as critical factors limiting sulfur utilization efficiency.

Fe atoms play distinct functional roles when coordinated with different metal partners. For instance, Fe mainly accelerates Li_2_S conversion in Fe─Co DACs. In Fe─Ni DACs, Fe exhibits superior capacity to capture long‐chain LiPSs and facilitate their LiPSs transformation, while Ni speeds up the redox kinetics of short‐chain LiPSs [142, 143]. Conventional carbon substrates facilitate electron transport to promote LiPSs conversion, yet the nonpolar adsorption and weak catalytic activity of carbon substrates lead to severe leakage of polar LiPSs. To address this issue, Fan et al. selected siloxane with outstanding electronic conductivity and favorable LiPSs affinity as the support, and systematically investigated the boosted LiPSs conversion kinetics after adsorpting a series of transition metal dual atoms (Mn, Fe, Co, Ni) [144]. Theoretical calculations indicate that siloxane molecules form strong orbital hybridization with metal d orbitals and enhance the interaction between LiPSs and TM_1_TM_2_ DACs. In particular, Fe‐Ni DACs form robust chemical bonds including Fe‐O, Ni‐O, Ni‐Si and Fe‐Si, ensuring structural stability of the DACs. The Fe‐Ni synergy minimizes the offset of d‐p orbitals in M─S bonds, accelerates S─S bond cleavage, achieves the lowest energy barrier (0.32 eV) for the conversion of Li_2_S_4_ to Li_2_S_2_ (Figure 9i). In comparison, the rate‐determining step barriers for Fe/Co/Ni SACs reach 0.65, 0.50 and 0.46 eV, respectively, demonstrating that orbital interactions between dual metal atoms can enable synergistic adsorption and catalysis, thereby improving the comprehensive electrocatalytic performance of DACs. Guided by the above theoretical findings, Kang et al. fabricated atomically dispersed Ni‐Fe DACs supported on 3D porous NC and verified that Ni atom catalyzes the transformation of long‐chain LiPSs, while Fe atom promotes Li_2_S nucleation [145]. In this case, due to the synergistic effect of Fe‐Ni dual atoms, the Ni‐Fe DACs realize rapid conversion of both long‐chain and short‐chain LiPSs with a reaction barrier of 1.21 eV (Figure 9j). Heteroatom doping induces local electron polarization to accelerate charge transport and enhances substrate‐polysulfide adsorption, enabling multi‐site adsorption of LiPSs. Accordingly, Zhu et al. introduced Ni‐Fe dual atoms together with N, F, B heteroatoms on NC to boost sulfur redox kinetics, and the corresponding Li─S cell delivers a high specific capacity of 1448 mAh g^−1^ at 0.2 C [146]. Differential charge density characterizations confirm that dispersed N, F and B heteroatoms shift the density of states of Ni‐Fe closer to the Fermi level, enabling rapid charge transfer between Ni‐Fe dual atoms and adsorbed Li_2_S_4_.

Beyond Fe, Co, Ni and other metal atoms serving as central active sites of DACs, Han et al. incorporated Si, a nonmetallic element with relatively high electronegativity, into Fe─N_4_ coordination structure to form Si‐Fe DACs [147, 148], which induces charge transfer from Fe to Si and elevates the overall catalytic activity of the Si‐Fe DACs. Accordingly, asymmetric coordination engineering combined with nonmetal heteroatom doping can synergistically modulate the 3d orbitals of Fe centers. Zhao et al. synthesized single‐atom catalysts with Fe─N_5_‐Te─N_4_ coordination supported on N‐doped carbon (FeTe/NC) [149]. The constructed coordination drives charge accumulation on Fe atoms, raises the energy levels of Fe d_xz_ and d_yz_ orbitals, and enlarges orbital overlap between Fe 3d and S 2p orbitals. As a result, the magnitude of charge transfer between Fe‐Te dual sites and LiPSs increases to 0.3 e^−^, compared with 0.2 e^−^ for Fe SACs (Figure 10a) [150]. Moreover, the introduction of Te strengthens the capture and catalytic conversion capacity of Fe atoms toward LiPSs, lowering the nucleation energy barrier of Li_2_S on FeTe/NC from 0.83 eV (for Fe/NC) to 0.63 eV (Figure 10b). Meanwhile, the decomposition barrier of Li_2_S (1.55 eV) and Li^+^ diffusion barrier (0.26 eV) on FeTe/NC are further reduced. In summary, nonmetal atom incorporation rearranges the charge distribution within dual single‐atom active sites, modulates the electronic structure and d‐band center of central Fe atoms, reinforces d‐p orbital hybridization, and delivers synergistic catalytic activity toward LiPSs (Figure 10c).

**FIGURE 10:**
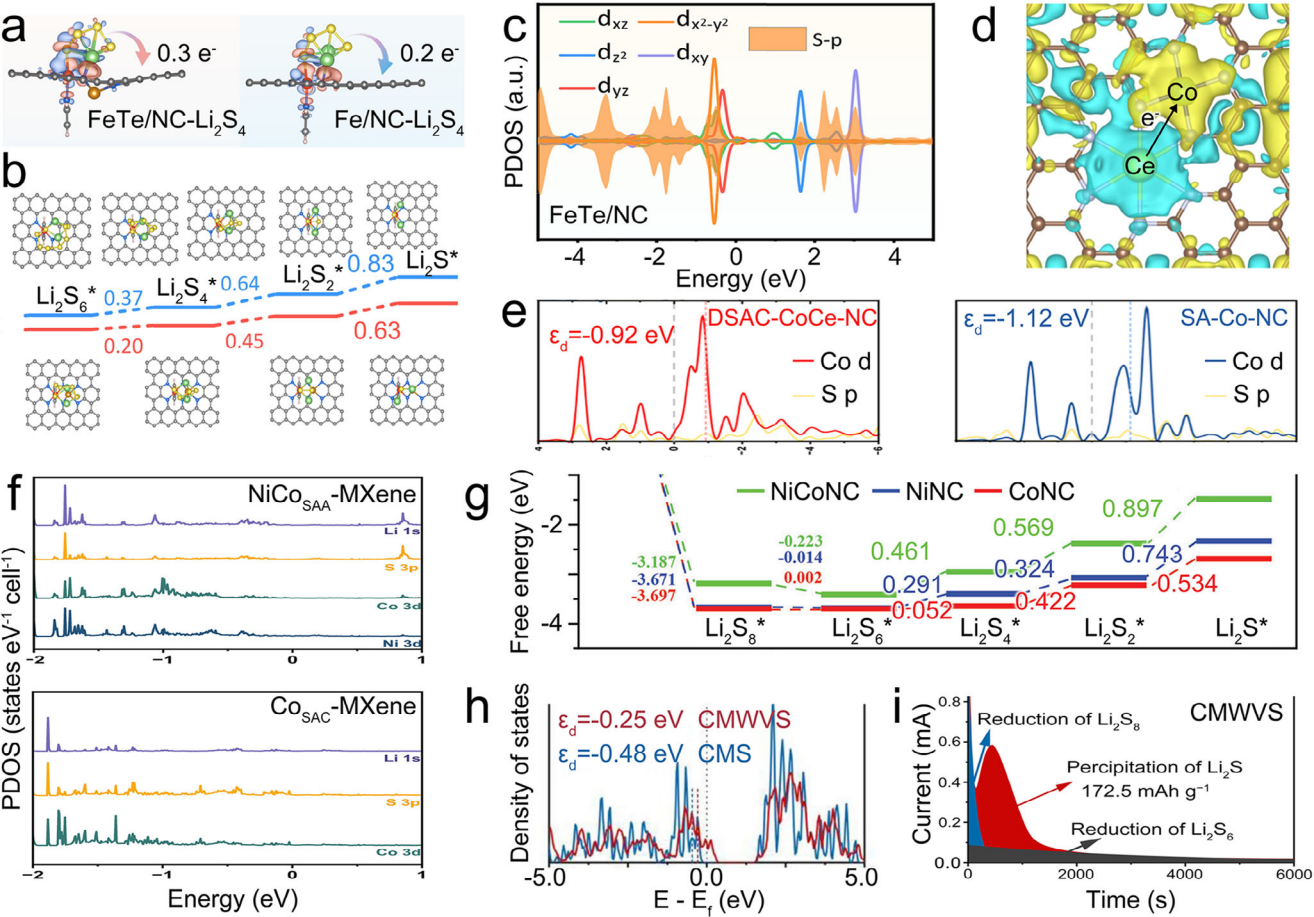
(a) Charge density difference of Li_2_S_4_‐FeTe/NC and Li_2_S_4_‐Fe/NC. (b) Free energy profiles for the conversion of LiPSs. (c) PDOS of Li_2_S_4_‐FeTe/NC. Reproduced with permission [149]. Copyright 2025, Springer Nature. (d) Charge density difference and (e) PDOS of Ce‐Co DACs. Reproduced with permission [152]. Copyright 2025, American Chemical Society. (f) PDOS of Ni‐Co DACs and Co SACs. Reproduced with permission [154]. Copyright 2025, Tsinghua University Press. (g) Free energy profiles for the conversion of LiPSs. Reproduced with permission [156]. Copyright 2024, Wiley‐VCH GmbH. (h) PDOS of CMWVS and CMS. (i) Li_2_S nucleation experiment. Reproduced with permission [157]. Copyright 2026, Springer Nature.

Although Fe sites effectively anchor long‐chain LiPSs and accelerate Li_2_S decomposition, the relatively low Li_2_S nucleation barrier of Fe atom leads to unsatisfactory overall LiPSs redox kinetics [151]. Rare‐earth cerium exhibits strong sulfur‐philicity, and the formation enthalpy of CeS (‐459.4 kJ mol^−1^) is lower than those of FeS_2_ (‐178.2 kJ mol^−1^) and Co_2_S_3_ (‐147.3 kJ mol^−1^). Such strong affinity enables Ce to firmly immobilize soluble LiPSs on the catalyst and expedite the conversion of long‐chain LiPSs [152, 153]. To complement Ce with rapid Li_2_S nucleation and dissolution capabilities, Co atoms were introduced to construct Ce‐Co DACs by Chen et al., which greatly improved sulfur utilization (92.8% after 1000 cycles at 0.5 C) [152]. Intermetallic charge transfer between Ce and Co increases the number of free electrons on the Co sites (+1.000 vs. +0.968), accelerates charge transport across Co─S bonds, and reduces the Gibbs free energy for the reduction of Li_2_S_2_ to Li_2_S to 0.70 eV (Figure 10d,e). Moreover, the Li_2_S nucleation response time on Co‐Ce DACs (201 s) is shorter than those on Co SACs (221 s) and Ce SACs (415 s), accompanied by the highest Li_2_S nucleation capacity of 149.8 mAh g^−1^. Benefiting from the synergistic adsorption and catalysis of Co and Ce dual sites, the Li_2_S dissolution time on CoCe DACs (1034 s) outperforms that on Co SACs (1138 s), with a high Li_2_S oxidation capacity of 338 mAh g^−1^. Therefore, screening metal centers with distinct functional roles to fabricate heteronuclear DACs realizes synergistic immobilization and transformation of long‐, medium‐ and short‐chain LiPSs. Both Ni and Co SACs possess favorable electrocatalytic activity yet weak adsorption affinity toward LiPSs. In our previous work, Co‐Ni SACs were immobilized on high‐specific‐surface‐area MXene substrates to suppress the LiPSs shuttling effect and accelerate LiPSs redox kinetics [154]. DFT calculations reveal that Co‐Ni bonding rearranges the electronic structure and induces intensive charge accumulation at the Fermi level of Co‐Ni DSCs, facilitating electron delocalization (Figure 10f) [155]. Thus, the synergistic formation of Ni‐S and Co─S bonds collectively accelerates LiPSs conversion. Consequently, Co‐Ni DACs/MXene‐based Li─S cell delivers a high discharge specific capacity of 746 mAh g^−1^ even at a high current rate of 4.0 C. To further advance the synergistic catalysis of Ni‐Co DACs, Zhang et al. fabricated DACs structures with Ni‐Co─N_6_ coordination supported on NC, which exhibits strong adsorption toward Li_2_S_8_, Li_2_S_4_ and Li_2_S_2_ with binding energies of ‐1.0, ‐1.1 and ‐1.77 eV, respectively [156]. As shown in Figure 10g, the Gibbs free energy for Li_2_S_2_ reduction on Ni‐Co─N_6_ (0.534 eV) is lower than those on monometallic Ni─N_4_ (0.897 eV) and Co─N_4_ (0.743 eV) SACs, demonstrating the thermodynamic superiority of dual‐site synergistic adsorption and catalysis for LiPSs reduction. Liu et al. adopted a hydrothermal method to grow V‐W co‐doped MoS_2_ nanosheets on carbon fiber substrates (CMWVS) [157]. The layered structure of CMWVS, with a large specific surface area, enables strong LiPSs adsorption, and abundant multi‐active sites expedite the conversion of anchored LiPSs. Besides, Co‐doping with V and W cooperatively modulates local electronic configurations and reduces the Li_2_S nucleation barrier. Owing to the low electronegativity of V, charge transfers toward the MoS_2_ substrate and strengthens the interaction between V sites and LiPSs. Meanwhile, the large atomic radius of W and intensified orbital coupling drive the phase transformation of MoS_2_ toward metallic character, shifting the d‐band center upward from ‐0.48 to ‐0.25 eV to accelerate surface charge transport and lower the LiPSs conversion barrier (Figure 10h). As shown in Figure 10i, W sites immobilize LiPSs via stable W─S bonding interactions and achieve the high Li_2_S nucleation capacity of 172.5 mAh g^−1^.

## 3 Conclusion and Perspectives

Li‐S batteries stand out as promising candidates for next‐generation energy storage systems owing to their ultrahigh energy density and low raw material costs. Nevertheless, the shuttling effect of soluble LiPSs, sluggish redox kinetics originating from multi‐step conversion and the intrinsic electronic insulation of S_8_ and Li_2_S collectively hinder the commercialization of Li─S batteries. Introducing highly active SACs to boost sulfur utilization by virtue of d‐p orbital hybridization between central metal atoms and sulfur species, as well as rapid charge transfer triggered by coordinating atoms, has become a vital research direction. In this review, we systematically summarize the mechanisms through which coordination chemistry environments and central metal screening enhance the catalytic activity of SACs (Figure 11). Firstly, the intrinsic limitations of catalytic performance for conventional symmetric M─N_4_ coordination structure are elaborated. Subsequently, we comprehensively discuss the related strategies including heteroatom‐induced asymmetric coordination, unsaturated coordination, axial coordination, and homonuclear/heteronuclear dual‐metal coordination modulate local charge distribution and orbital hybridization to increase the catalytic efficiency (Table 1, 2). However, in the future, the development of coordination chemistry on SACs is prospected to accelerate the electrochemical kinetics.

1. Although the symmetric M─N_4_ coordination structure delivers excellent structural robustness and moderate catalytic activity, the charge of the metal center is tightly confined within the planar M─N_4_ coordination structure by the highly electronegative N atoms, which weakens *d‐p* orbital overlap between metal sites and sulfur species, restricts the charge delocalization radius and suppresses catalytic activity. Meanwhile, orbital hybridization between the metal and nitrogen reduces the degree of *d‐p* orbital overlap between metal and sulfur, slowing down interfacial charge transfer. Accordingly, breaking the stable M─N_4_ coordination to generate uneven charge distribution is essential to strengthen M‐S interactions and accelerate LiPSs conversion kinetics. Incorporating nonmetallic coordinating atoms with low electronegativity, abundant valence electrons and large atomic radii (such as P and S) can form metastable M─N_3_X configurations, increasing the total charge density of single‐atom active sites and inducing unidirectional electron cloud polarization to realize delocalized charge sharing. In addition, the surplus valence electrons of P and S occupy empty metal orbitals, switching the metal spin state from low spin to high spin and further accelerating charge transfer across M─S bonds. Nevertheless, precise synthetic control over target asymmetric coordination structure remains experimentally challenging. Thus, the future synthetic methodologies should aim for high‐fidelity construction of well‐defined metastable coordination configurations instead of mixed disordered active sites.
2. The electron transport pathway of LiPSs adsorbed on planar M─N_4_ sites is confined to a 2D plane. Therefore, introducing axial coordinating atoms or large‐radius coordinating atoms during structural design can trigger geometric distortion of SACs, driving charge diffusion from the 2D plane into 3D space and expanding the charge‐sharing radius along the z‐axis. Structural distortion also elongates M─S bonds, facilitates the cleavage of S─S bonds, and lowers the overall conversion energy barrier of LiPSs. However, experimentally obtaining well‐controlled axially coordinated SACs with uniform axial ligands remains challenging. Advanced synthetic strategies are urgently required to precisely tailor axial ligand species, bond lengths, and distortion degrees to harness the 3D charge modulation capability of axial coordination.
3. The central metal atoms undertake core functions of LiPSs anchoring and charge transport throughout LiPSs redox processes. However, a single type of metal only exhibits strong affinity toward either long‐chain or short‐chain LiPSs, rendering monometallic SACs inefficient for the full cascade conversion from Li_2_S_8_ to Li_2_S. To address this limitation, artificial intelligence combined with high‐throughput computation can be adopted to screen multiple metal species, followed by experimental verification to construct atomically dispersed polymetallic catalysts. The synergistic effect of multiple metal centers enables simultaneous capture and rapid transformation of LiPSs. Electronegativity differences between heteronuclear metal atoms induce electron cloud polarization, boosting the catalytic activity of the more electronegative metal sites to expedite LiPSs redox. Electron‐deficient metal atoms provide abundant empty orbitals, amplifying d‐p orbital overlap between metal and sulfur for stronger LiPSs immobilization. Furthermore, large atomic radii of metal centers distort the geometry of active sites, enabling 3D delocalized charge sharing and accelerating LiPSs conversion kinetics.
4. The coordination number and precise local coordination structure of SACs cannot be unambiguously determined by any single characterization technique. High‐angle annular dark‐field scanning transmission electron microscopy (HAADF‐STEM) primarily verifies the atomic dispersion of metal centers and offers spatial distribution information, but it fails to resolve bond lengths, coordination numbers or detailed ligand configurations. X‐ray absorption near‐edge structure (XANES) spectroscopy reflects the oxidation state and qualitative coordination features of metal sites. Extended X‐ray absorption fine structure (EXAFS) spectroscopy together with wavelet‐transform EXAFS (WT‐EXAFS), affords quantitative structural parameters including bond length and coordination number. Nevertheless, each technique has inherent limitations and typically yields statistically averaged information. Accordingly, the real coordination configuration of SACs requires a synergistic combining multiple characterizations with theoretical calculations to screen plausible structural models. Meanwhile, the real time variation of the coordination and electronic configurations of SACs during the transformation of LiPSs in electrocatalytic processes are still ambiguous. Hence, operando characterizations under working electrochemical conditions are required to clarify the intrinsic structure‐activity correlation between local coordination environment and catalytic activity.
5. Current theoretical calculations for SACs are mostly performed on highly idealized models, which fail to accurately reflect the actual coordination environments and spatial geometries of SACs synthesized in experiments. Moreover, atomic‐level dispersion leads to inconsistent local environments across active sites, creating a prominent discrepancy between theoretical predictions and practical electrochemical performance. In addition, DFT calculations are generally carried out under static vacuum conditions, ignoring dynamic variations in adsorption and catalytic behavior induced by real electrocatalytic operating factors, including electrolyte components, solvent molecules, LiPSs concentration gradients, external electric fields and temperature fluctuations. To bridge the gap between theoretical simulation and experimental practice and to guide catalyst design more effectively, more realistic computational frameworks should be established by incorporating external electric fields, electrolyte parameters and multi‐state structural models to identify optimal catalytic configurations.

**FIGURE 11:**
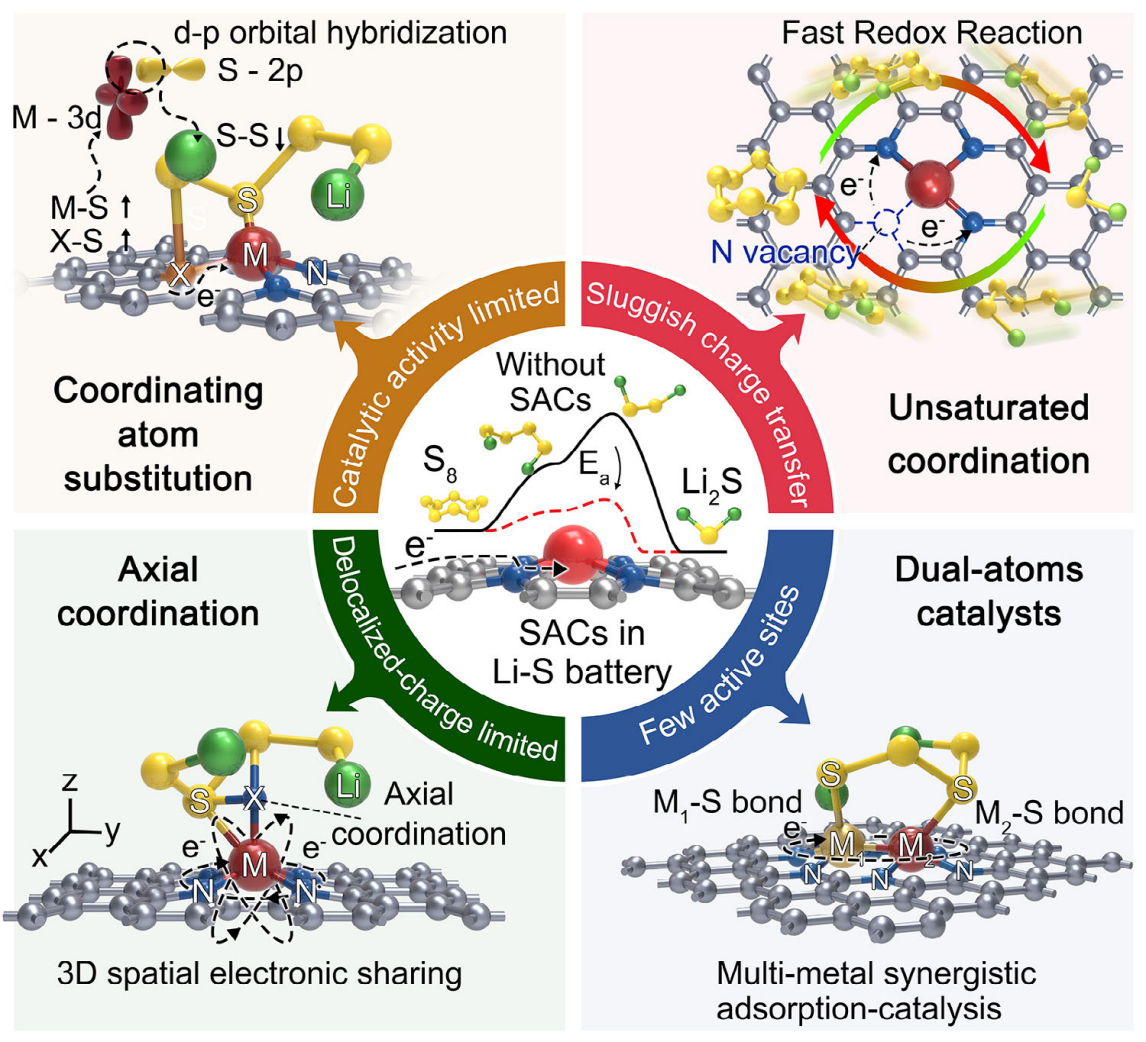
Design logic of metastable SACs coordination.

**TABLE 1:** Summary of coordination configuration‐electronic structure‐kinetics of partial SACs in this review.

Coordination structure	Electronic structure	Adsorption energy	Redox energy barrier	Ref
Co─N_3_B	*d‐p* orbital hybridization	1.65 eV (Li_2_S_4_)	0.66 eV (Li_2_S)	[65]
Co─N_2_C_2_	electron density shift toward N		0.43 eV (Li_2_S)	[69]
Co─N_1_C_3_	d‐band center upward (‐0.34 eV)		1.01 eV (Li_2_S)	[71]
Sm─N_3_C_3_	*4f‐5d‐2p* orbital coupling		0.72 eV (Li_2_S)	[72]
Co─N_2_O_2_	electron density shift toward O		0.784 eV (Li_2_S)	[73]
Cu─N_3_F	dynamically modulate the electron distribution		0.19 eV (LiPSs)	[76]
Sc@N_3_P_1_	more free electron & electron delocalization		1.22 eV (LiPSs)	[80]
Co─N_2_P_2_	electron aggregation & conformational distortion		1.65 eV (Li_2_S)	[86]
Co─N_1_S_3_	reduce the electron density			[95]
Fe─N_3_S_1_	trigger electron redistribution	−1.11 eV (LiPSs)		[98]
Mn─N_3_Cl	narrow the band gap		1.67 (LiPSs)	[106]
Co─N_3_	Intensified *d‐p* orbital hybridization	2.37 eV (Li_2_S_6_)	1.22 eV (Li_2_S)	[111]
Co─N_4_‐O_2_	promot the delocalization of free electron		0.62 eV (Li_2_S)	[117]
2Fe─N_6_	2Fe sites synergistically anchor & catalytic		1.21 eV (LiPSs)	[123]
CuFe DACs	prominent *d‐p* orbital hybridization	1.31 eV (LiPSs)	0.97 eV (Li_2_S), 0.65 eV (Li_2_S_2_)	[131]
NiFe DACs	prominent *d‐p* orbital hybridization		0.32 eV (Li_2_S)	[144]
NiCo DACs	charge accumulation & electron delocalization	−1.77 eV (Li_2_S)	0.534 eV (Li_2_S_2_)	[156]

**TABLE 2:** Summary of coordination configuration‐electrochemical performance of partial SACs in this review.

Coordination structure	Sulfur loading	Catalyst loading	N/P ratio	Current density	Electrochemical performance	Ref
Co─N_3_B	1.0 mg cm^−2^		16 µL mg^−1^	1.0 C	615.1 mAh g^−1^ (500^th^)	[65]
Co─N_2_C_2_	6.1 mg cm^−2^	∼0.15 g cm^−2^	20 µL mg^−1^ (cell)	0.1 C	6.85 mAh cm^−2^	[69]
Co─N_1_C_3_	9.6 mg cm^−2^		4.2 µL mg^−1^	0.1 C	6.32 mAh cm^−2^ (80^th^)	[71]
Sm─N_3_C_3_	5.2 mg cm^−2^		6.4 µL mg^−1^	1.0 C	71% (100^th^)	[72]
Co─N_2_O_2_	5.6 mg cm^−2^	∼1.1 mg cm^−2^	5.1 µL mg^−1^	2.0 C	5.98 mAh cm^−2^	[73]
Co─N_2_P_2_	3.01 mg cm^−2^	∼0.14 mg cm^−2^	∼5 µL mg^−1^	0.1 C	1004.3 mAh g^−1^ (1^st^)	[86]
Fe‐P_4_	5.7 mg cm^−2^		6.2 µL mg^−1^	0.1 C	88.3% (100^th^)	[93]
Co─N_3_S_1_	1.5‐2.0 mg cm^−2^		40 µL	1.0 C	99.24% (1000^th^)	[95]
Fe─N_3_S_1_	6.0 mg cm^−2^	∼0.4 mg cm^−2^		0.2 C	4.3 mAh cm^−2^ (300^th^)	[98]
Mn─N_3_Cl	2.3 mg cm^−2^	∼0.7 mg cm^−2^	15 µL mg^−1^	0.2 C	894.5 mAh g^−1^ (100^th^)	[105]
Co─N_3_	3.8 mg cm^−2^			1.5 C	845 5 mAh g^−1^ (300^th^)	[111]
Co─N_4_‐O_2_	2.5 mg cm^−2^	∼7:3 (S/C)	10 µL mg^−1^	1.0 C	460 mAh g^−1^ (500^th^)	[117]
2Fe─N_6_	21.8 mg cm^−2^	∼4:1 (S/C)	5 µL mg^−1^	0.2 C	23.8 mAh cm^−2^ (1^st^)	[123]
2Cu DACs	7.7 mg cm^−2^		4.8 µL mg^−1^	1.0 C	7.8 mAh cm^−2^ (1^st^)	[127]
ZnBi DACs	∼1.2 mg cm^−2^	∼3:1 (S/C)	20 µL mg^−1^	1.0 C	81% (950^th^)	[135]
NiFe DACs	∼6 mg cm^−2^	∼4:1 (S/C)	8 µL mg^−1^	0.2 C	629 mAh g^−1^ (50^th^)	[145]
NiCo DACs	4.5 mg cm^−2^		8 µL mg^−1^	0.2 C	494.2 mAh g^−1^ (200^th^)	[156]
FeTe DACs	8.7 mg cm^−2^	∼0.5 mg cm^−2^	4.9 µL mg^−1^	1.0 C	5.6 mAh cm^−2^ (50^th^)	[149]

In summary, we believe that rationally designed metastable SACs coordination structures with superior intrinsic catalytic activity can fully unlock the performance advantages of Li─S batteries. Combining the low cost and high catalytic activity of SACs with the high capacity and low raw material cost of sulfur cathodes will greatly promote the commercialization progress of Li─S batteries energy storage technology.

## Author Contributions

C. Peng and J.Y. Zhao conceived the idea and wrote the manuscript. C. Peng and J. Zhang designed the figure. J. Wang, H. Li, W. Li, Y. Miao and Y. Zhang modified the review. J. Zhang and, J. Wang, provided the funding.

## Conflicts of Interest

The authors declare no conflicts of interest.

## Data Availability

No data is generated in this work.
